# How Do Cancer-Related Mutations Affect the Oligomerisation State of the p53 Tetramerisation Domain?

**DOI:** 10.3390/cimb45060317

**Published:** 2023-06-07

**Authors:** Federica Nicolini, Toni Todorovski, Eduard Puig, Mireia Díaz-Lobo, Marta Vilaseca, Jesús García, David Andreu, Ernest Giralt

**Affiliations:** 1 Institute for Research in Biomedicine (IRB Barcelona), Baldiri Reixac 10, 08028 Barcelona, Spain; federica.nicolini@irbbarcelona.org (F.N.); toni.todorovski@uniri.hr (T.T.); epuig@scripps.edu (E.P.); mireia.diaz@irbbarcelona.org (M.D.-L.); marta.vilaseca@irbbarcelona.org (M.V.); jesus.garcia@irbbarcelona.org (J.G.); 2 Department of Medicine and Life Sciences, Universitat Pompeu Fabra, Barcelona Biomedical Research Park, Dr. Aiguader 88, 08003 Barcelona, Spain; david.andreu@upf.edu; 3 Department of Inorganic and Organic Chemistry, University of Barcelona, Martí i Franquès 1-11, 08028 Barcelona, Spain

**Keywords:** p53, p53 tetramerisation domain, cancer mutations, native MS, high-field NMR, diffusion NMR, p53 tetramer, p53 dimer

## Abstract

Tumour suppressor p53 plays a key role in the development of cancer and has therefore been widely studied in recent decades. While it is well known that p53 is biologically active as a tetramer, the tetramerisation mechanism is still not completely understood. p53 is mutated in nearly 50% of cancers, and mutations can alter the oligomeric state of the protein, having an impact on the biological function of the protein and on cell fate decisions. Here, we describe the effects of a number of representative cancer-related mutations on tetramerisation domain (TD) oligomerisation defining a peptide length that permits having a folded and structured domain, thus avoiding the effect of the flanking regions and the net charges at the *N*- and *C*-terminus. These peptides have been studied under different experimental conditions. We have applied a variety of techniques, including circular dichroism (CD), native mass spectrometry (MS) and high-field solution NMR. Native MS allows us to detect the native state of complexes maintaining the peptide complexes intact in the gas phase; the secondary and quaternary structures were analysed in solution by NMR, and the oligomeric forms were assigned by diffusion NMR experiments. A significant destabilising effect and a variable monomer population were observed for all the mutants studied.

## 1. Introduction

The transcription factor p53 is a tumour suppressor that plays a crucial role in many biological processes. It is called “the genome guardian” due to its ability to bind to DNA and check its integrity [1]. In response to stress, p53 trans-activates a variety of genes by binding to specific DNA sequences and promoting the repair of damaged DNA, as well as promoting cell cycle arrest and apoptosis [2]. It contributes to genome protection and also favours senescence in order to maintain genome stability [3].

The activity of p53 is very precisely regulated by factors such as stability, cellular localisation and tetramerisation. In healthy cells, p53 expression is low [4], and the protein is located mainly in the cytosol. It is unstable, with a half-life between 20 and 50 min, and its oligomeric state is still under investigation [5]. Several studies reported that, in unstressed conditions, the protein is mainly dimeric [6]. Fluorescence correlation spectroscopy (FCS) experiments in vitro [5] and in cells [7] consistently point to the dimer as the most abundant oligomer, although large cell-to-cell variation has been reported in cell assays [7]. It has also been proposed that different oligomeric species of p53 coexist in solution; i.e., monomers, dimers and tetramers are in equilibrium [5]. Factors that damage DNA, such as hypoxia, telomere shortening and oncogene activation, induce posttranslational modifications that rapidly stabilise p53 [5,8,9] and increase its concentration, shifting the equilibrium towards tetramers. Tetramerisation enhances the affinity of the protein to specific DNA sequences [10] and, as a consequence, its transcriptional activity. Although it is universally recognised that p53 acts as a homo-tetramer protein, the tetramerisation mechanism is not fully understood. Various models have been proposed describing an interaction between the *C*- and *N*-terminus in murine p53 as key interactions for the tetramerisation of the protein [11]. Nevertheless, for human p53, the model suggests that tetramerisation occurs prevalently due to the presence of the tetramerisation domain (TD) [12,13]. The oligomerisation of p53 may occur stepwise in different times and areas of the cell. In particular, dimerisation occurs co-translationally on the polysome, whereas tetramerisation is a posttranslational event that takes place in the nucleus [14,15]. Appropriate subcellular localisation is modulated by specific sequences within the *C*-terminal region [16,17]. In particular, there are three nuclear localization sequences (NLSs) (residues 316–325, 369–375 and 379–384) and a nuclear exporting sequence (NES) (residues 340–351), the latter being buried when the protein is in its tetrameric form [18] but accessible in the monomeric or dimeric forms. The interaction of p53 with a broad number of proteins constitutes an additional layer in the regulation of p53 oligomerisation. Proteins such as RhoGAPs, BCCIP, 14-3-3 γ and ε, Atg7, and MYBBP1A stabilise the tetramer, whereas RBEL1A, S100, and ARC prevent tetramerisation [19,20,21,22,23,24,25,26]. Notably, the E3 ubiquitin ligase, MDM2, preferentially binds and degrades p53 in its dimeric form [27].

However, in spite of this complex regulatory network, mutations can dramatically affect the oligomeric state of p53. Cancer onset is frequently associated with p53 mutations, and most of these cancer-related mutations occur in the DNA-binding domain (DBD). These mutations alter the interaction between p53 and DNA, although they can also modulate the aggregation state of the protein [28]. Notably, ca. 20% of cancer-associated mutations occur in the TD, being almost six times smaller in length than the DBD (TD ~35 residues vs. DBD ~200 residues) [29,30,31]. However, it is still unclear how the altered activity of mutated p53 is related to cancer development. It has been suggested that mutant p53 acts as a guardian of cancer cells by protecting them against metabolic pathway rewiring, which is normally activated in response to stress [32]. Numerous studies have established that cancer-related mutations within the TD lead to a decrease in transcriptional activity in cells that correlate with the destabilisation of the p53 quaternary structure [2,31,33,34,35]. These studies showed that TD mutations were detrimental for tetramerisation, but most were based on overexpression experiments and only provided a semi-quantitative analysis of p53 oligomerisation. For this reason, Kamada et al. used gel filtration chromatography to characterise in a systematic study the effect of 49 cancer-associated mutations on the oligomerisation of TD peptides [29], observing that most mutations destabilise the tetrameric structure. Since then, there has been a resurgence of interest in the link between the functional activity and the oligomerisation status of p53 [13,36,37]. Fischer and co-workers reported that the oligomerisation of p53 is crucial for cell fate decisions regarding growth, cell cycle arrest and apoptosis [36]. Mutating several TD positions, the authors demonstrated that tetramerisation is required to induce apoptosis, but the dimer is still able to arrest cell growth.

This work delves into the influence of cancer related mutations on the oligomeric state of p53 TD. Although different groups focused on this topic previously, they worked with different lengths of the peptides, some of them obtained by chemical synthesis and others by expression in *E. coli*, and they applied different techniques to determine the oligomeric states of the TD, mainly gel filtration chromatography [29] and NMR [12]. We propose a set of data, where the TD peptide has been defined to obtain a folded domain, avoiding the effect of the flanking region [38]. Moreover, our peptides were obtained in their acetylated and amidated form in the *N*- and *C*-terminus, respectively, to avoid the net charges mimicking the amide bond at the extremes. We have selected a set of representative mutations (T329I, R337H, R342L, L344R, L344P, D352H) to address their effect on the secondary and quaternary structures of TD under different experimental conditions. For this purpose, we have used a variety of biophysical techniques, such as circular dichroism (CD), native MS and high-field solution NMR. Native MS allowed us to determine the quaternary structure of our peptides in the gas phase, maintaining the structure of the complexes as in solution. Then, our systems were studied also in solution, by HSQC NMR and diffusion NMR experiments to assign the oligomeric state of our peptides. As expected, the WT TD assembles as a tetramer. On the contrary, a significant population of monomer was observed for all the mutants studied regardless of the major oligomeric species of the mutant (tetramer or dimer), the secondary structure and the solvent exposure of the mutated position.

## 2. Materials and Methods

### 2.1. Synthesis of the 37TDs

The peptides of interest were synthesised in their acetylated (at *N*-terminus) and amidated (at *C*-terminus) forms by Fmoc/*t*Bu solid-phase peptide synthesis (SPPS). The peptides consist of L-amino acids, and the isotope-labelled sequences were obtained using ^13^C-methyl methionine provided by Cambridge Isotope Laboratories (Tewksbury, MA, USA).

**Figure 1:**
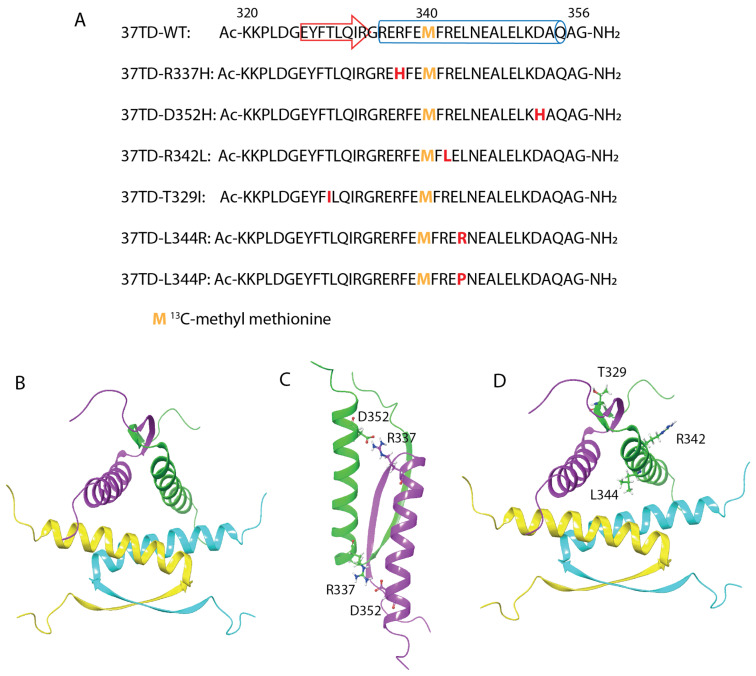
(**A**) Sequences of the 37TDs. The red residues correspond to the positions of the mutations, and the yellow Met indicates the isotope-labelled residue. The β-strain and the α-helix are represented with an arrow and a cylinder, respectively, in the WT sequence. For 37TD-WT and 37TD-R337H, only the isotope-labelled sequences are shown. (**B**) Cartoon representation of the WT (dimer of dimers) from its NMR structure [54], PDB ID 1OLG. (**C**) Cartoon representation of the dimer showing location of the salt bridge between R337 and D352. (**D**) Cartoon representation of the WT showing location, only in one monomer, of the residues that are mutated (T329, R342, and L344).

37TD-WT and 37TD-R337H were synthesised manually on H-Rink Amide ChemMatrix resin at 0.1 mmol scale. The resin was conditioned by washing with MeOH (5 × 30 s), DMF (5 × 30 s), DCM + 1% TFA (2 × 10 min), DCM (5 × 30 s), DMF (5 × 30 s), DCM (5 × 30 s), DCM + 5% DIPEA (2 × 10 min), DCM (5 × 30 s) and DMF (5 × 30 s) using 10 mL per gram of resin each time. The first amino acid was attached twice using distinct coupling conditions. The first time, the protected amino acid (3 eq.) was activated using diisopropylcarbodimide (DIC, 3 eq.) and Oxyma Pure (3 eq.), and the second time, it was activated using HATU (3 eq.) and DIPEA (6 eq.). The mixture was allowed to react in an orbital shaker at room temperature (RT) for 1 h and 40 min, respectively. The yield of this reaction was calculated by UV absorption of the dibenzofulvane, the leaving group of Fmoc, at 301 nm [39,40]. The remaining active sites on the resin that did not react with the first amino acid (free NH_2_ groups) were capped through acetylation with a solution of 5% acetic anhydride, 8.5% DIPEA, and 86.5% DMF, by stirring at RT for 15 min. The chain up to the 11th residue was elongated using HATU/DIPEA as coupling reagents, followed by DIC/Oxyma Pure until the end of the synthesis. The efficiency of each coupling step was followed by the Kaiser (for primary amines) [41] and chloranil (for secondary amines) tests [42]. The Fmoc group was removed using two solutions of 40% and 20% (*v*/*v*) of piperidine in DMF until deprotection of the G325 was achieved (Figure 1A), and then a solution of 10% (*w*/*v*) piperazine in 90:10 NMP in EtOH with 0.1 M Oxyma Pure was used in order to prevent the formation of the aspartimide, which is particularly prone after the DG sequence [43]. 37TD-WT and 37TD-R337H were synthesised twice, using a Fmoc-methionine or ^13^C-methyl methionine, respectively. 

The other 37TDs (37TD-D352H, 37TD-R342L, 37TD-T329I, 37TD-L344R, and 37TD-L344P) were synthesised using a Liberty Blue CEM microwave-assisted peptide synthesiser (Matthews, NC, USA). All these sequences were obtained using a ^13^C-methyl methionine and were synthesised on Rink Amide ProTide LL resin, on a reaction scale of 0.05 mmol. The resin was conditioned with DCM for 15 min and afterwards placed in the synthesiser without preloading of the first amino acid. Each coupling was performed using 4 eq. of the Fmoc-amino acid and DIC (4 eq.)/Oxyma Pure (4 eq.) in DMF. All the couplings were programmed as double coupling, apart from the introduction of R, K, E, and M, which were set up as triple coupling. The Fmoc group was removed with a solution of 10% (*w*/*v*) piperazine in 90:10 NMP in EtOH containing 0.1 M Oxyma Pure.

All synthesised peptides were acetylated at their *N*-terminus using a solution of 5% acetic anhydride, 8.5% DIPEA, and 86.5% DMF in an orbital oscillator at RT for 15 min.

The 37TDs that were manually synthesised were cleaved from the resin and deprotected with a cocktail of 92.5% TFA, 2.5% H_2_O, 2.5% triisopropylsilane (TIPS) and 2.5% dithiothreitol (DTT), while for the rest of the sequences a cocktail of 92.5% TFA, 2.5% H_2_O, 2.5% TIPS and 2.5% 2,2′-(Ethylenedioxy)diethanethiol (EDT) was used. In both cases, the reaction was left for 3 h, and the crude peptides were precipitated with cold Et_2_O, and then they were solubilised in water and lyophilised. 

#### 2.1.1. Peptide Purification

37TD-WT and 37TD-R337H were purified by semi-preparative HPLC on a Waters system (Milford, MA, USA) with MassLynx software, a 2545 binary gradient module, a 2767 manager collector, and a 2998 photodiode array detector, using Aeris C_18_ column (250 × 10 mm, 5 µm, 100 Å, Phenomenex, Torrance, CA, USA). The lyophilised peptides were dissolved in 5% acetonitrile in water and 9 mL of the solution was injected. The flow rate was 6.6 mL/min, solvent A = 0.1% TFA in water, solvent B = 0.1% TFA in acetonitrile. Elution was carried out with linear 10–45% gradients of solvent B into A over 30 min, with UV detection at 220 nm.

The other 37TDs were purified by semi-preparative HPLC on a Shimadzu LC-8A system (Kyoto, Japan) with UV detection at 220 nm and an Aeris C_18_ column (250 × 10 mm, 5 µm, 100 Å, Phenomenex, Torrance, CA, USA). The lyophilised peptides were dissolved in 5% acetonitrile in water, and 9 mL of solution was injected. The flow rate was 5 mL/min, solvent A = 0.1% TFA in water and solvent B = 0.1% TFA in acetonitrile. Elution was carried out with linear 5–60% gradients of solvent B into A over 30 min, with UV detection at 220 nm.

Fractions corresponding to the pure peptides (>85%) were collected, combined, lyophilised and stored at −20 °C.

#### 2.1.2. Characterisation

The manually synthesised and purified peptides (37TD-WT and 37TD-R337H) were characterised by UPLC and UPLC-MS. UPLC analysis was performed on a Waters Acquity equipped with Acquity photodiode array detector, using flow rate of 0.610 mL/min, Acquity UPLC BEH C_18_ column, 130 Å, 1.7 µm, 2.1 mm × 100 mm, solvents A = 0.045% TFA in water, and B = 0.036% TFA in acetonitrile. Elution was carried out with linear 0–70% gradients of solvent B into A over 40 min. UPLC-MS analysis was performed on a Waters Acquity UPLC System equipped with ESI-SQ Detector2, using a flow rate of 0.610 mL/min, Acquity UPLC BEH C_18_ column, 130 Å, 1.7 µm, 2.1 mm × 100 mm, solvents A = 0.1% formic acid in water and B = 0.07% formic acid in acetonitrile. Elution was carried out with linear 0–70% gradients of solvent B into A over 40 min. 

The other 37TDs were characterised by HPLC and HPLC-MS. HPLC analysis was performed on a C_18_ column, 4.6 mm × 50 mm, 3 µm, Phenomenex, Torrance, CA, USA in a LC-2010A system (Shimadzu, Kyoto, Japan), solvents A = 0.045% TFA in water, and B = 0.036% TFA in acetonitrile. Elution was carried out with linear 5–60% gradients of solvent B into A over 15 min. HPLC-MS analysis was performed on C_18_ column, 4.6 mm × 150 mm, 3.5 µm, Phenomenex, Torrance, CA, USA in a Shimadzu LC-MS 2010EV instrument, solvents A = 0.1% formic acid in water, and B = 0.08% formic acid in acetonitrile. Elution was carried out with linear 5–60% gradients of solvent B into A over 15 min.

### 2.2. Circular Dichroism

The lyophilised peptides were dissolved either in 50 mM sodium phosphate buffer, pH 7, or in water, pH 7, to a final monomer concentration of 20 µM. The spectra were recorded in a Jasco J-810 spectropolarimeter equipped with a Jasco-CDF-426S Peltier thermostatted cell holder and a Julabo external bath. Each CD spectrum was obtained by averaging three scans, recorded at 10 nm/min, DIT of 4 s, band width 1 nm, T = 25 °C and λ 190–260 nm, using a 1 mm path length quartz cell. Experimental data were smoothed using the software package provided by Jasco.

### 2.3. Native Mass Spectrometry

MS samples were prepared by dissolving lyophilised peptides in 200 mM ammonium acetate buffer, pH 7, to a final monomer concentration of 100 µM. The samples were further cleaned from salts by centrifugation in a Viva spin 500 with 3 kDa cut off (11,500 rpm, 4 °C, 20 min, three times). The final concentration was estimated by NanoDrop at 280 nm. The samples were stored in ice during the acquisition. Experiments were performed on a Synapt G1 HDMS (Waters, Milford, MA, USA) equipped with an Advion TriVersa NanoMate (Advion Biosciences, Ithaca, NY, USA). The positive mode for ESI was used. The source temperature, voltages and parameters are summarised in Table 1. Acquisitions were performed in the *m*/*z* range of 800–7000 with a 1.5 s scan time. Spectra were smoothed (smoothing method, mean; smooth window, 3; number of smooths, 2) with MassLynx V4.1 software (Waters). Definitive voltages for the capillary and the cone were tuned according to the threshold values (Table 1). These parameters were optimized to 1.5–1.75 kV and 20 V for the capillary and the cone, respectively. In order to facilitate ion transmission in the trap region, collision energy (CE) and bias were kept at the minimum value (6 V and 15 V, respectively); higher values caused unfolding of gaseous protein ions [44]. Finally, the backing pressure was minimised in order to avoid possible structural interactions of POP gaseous ions [45]; lower values than those recommended by Ruotolo et al. were chosen [46]. In addition, all these voltages and pressures were compatible for native calibrating protein ions.

The ion mobilograms were analysed using DriftScope 2.5 software and displayed with *m*/*z* versus ms.

### 2.4. Nuclear Magnetic Resonance (NMR)

NMR spectra were recorded on a Bruker 600 MHz spectrometer equipped with a TCI cryoprobe. The NMR samples were prepared by dissolving the lyophilised peptides in D_2_O at a final monomer concentration of 100 µM. The pD was adjusted using the equation [47]:

pD = pH + 0.4

Chemical shifts were referenced to internal DSS (4,4-dimethyl-4-silapentane-1-sulfonic acid) at 0.0 ppm. The HSQC of 37TDs were recorded at different temperatures, i.e., 25, 5, 37 or 40 °C.

Diffusion NMR experiments of 37TDs (100 μM) were performed at 25 °C using an in-house modified X-STE pulse sequence [48] to measure the diffusion of ^13^C-attached ^1^H. Encoding/decoding gradient lengths (δ) of 2.7–3.0 ms and diffusion delays (Δ) of 100–120 ms were used. Diffusion measurements under identical experimental conditions were carried out for 1,4-dioxane. In this case, the PG-SLED sequence [49] was used applying a gradient time (δ) of 1.8 ms and a diffusion time (Δ) of 70 ms. Diffusion coefficients (D^37TD^ and D^diox^) were obtained fitting the gradient strength-dependent decay in signal intensity to a mono exponential equation using the MestreNova 14.0.1 software.

Hydrodynamic radii of 37TDs (R_H_^37TD^) were deduced using the following equation:

R_H_^37TD^ = (D^diox^/D^37TD^) × R_H_^diox^

assuming that R_H_^diox^ is 2.12 Å [49].

Predicted hydrodynamic radii were calculated from empirical equations for folded proteins [50]:

R_H_ = 4.75 × N^0.29^

and for disordered proteins:

R_H_ = (1.24 × P_Pro_ + 0.904) × (0.00759 × |Q| + 0.963) × 2.49 × N^0.509^

where N is the number of residues (37, 74, and 148 for monomer, dimer, and tetramer, respectively), P_Pro_ is the fraction of proline residues, and |Q| is the absolute net charge. |Q| was calculated at pH 7 using the Protein Calculator v3.4 software (http://protcalc.sourceforge.net (accessed on 15 May 2021)).

## 3. Results and Discussion

### 3.1. Definition of the TD and Selection of the Mutations

The definition of the p53 tetramerisation domain (TD) differs slightly in the literature (Table 2). 

For all the peptides described in this study, we considered the TD as being between residues 320 and 356 [52,53]. These peptides are 37 amino acids long and are named 37TDs. In the wild-type TD, four monomers assemble to form a dimer of dimers (Figure 1B) [54]. Each monomer has a V-shape and is composed of a β-strain (res 326–333), a turn (G334) and an α-helix (res 335–354). Several interactions stabilise first the dimer and the tetramer as well. The salt bridge between R337 and D352 is an essential stabilising interaction of the dimer (Figure 1C). Hydrophobic interactions at the dimer–dimer interphase between side chains of the residues composing the α-helix stabilise the tetramer. We selected the wild-type (37TD-WT, Figure 1A,B) and six mutations, all of them related to cancer [29], to study the equilibrium between oligomers (Figure 1). The chosen mutations involve the residues of the salt bridge (R337H and D352H), residues on the α-helix (R342L, L344R and L344P) and a residue on the β-strain (T329I). Both residues forming the salt bridge were mutated in order to study the role of the salt bridge in the equilibrium between oligomers. R337H is the most common mutation in the TD, and it is associated with Li-Fraumeni syndrome, which gives rise to several types of cancer. Due to its correlation with cancer, this mutant has been studied and characterised by several groups [55,56,57,58,59], as well as in yeast [15] and mouse [60] models. Di Giammarino and co-workers reported that R337H strongly destabilises the tetramer and that its stability is pH-dependent, i.e., tetramer stability is partially recovered by decreasing the pH to acidic values [55]. In the presence of the D352H mutation, the negatively charged D352 residue from the salt bridge R337-D352 is replaced by a His residue. Here, the stability of the tetramer could still depend on the pH, as a His residue has been introduced. However, in this case, by protonating the imidazole group, two positive charges (from R337 and H352) would interact, reducing the stability of the tetramer. We chose a mutation in the α-helix, R342L, to destabilise the α-helix by disrupting the side chain interactions of R342 with E339 and E346 (*i, i − 3* and *i, i + 4*). Moreover, R342L is the second most common mutation in the TD. The mutation T329I in the β-strain in principle does not destabilise the structure of the tetramer; on the contrary, it could enhance the thermal stability of the domain compared to the WT (Figure 1A,D) [29]. Finally, L344R and L344P were introduced since it has been proposed that they promote the monomeric or dimeric forms, respectively (Figure 1A,D) [29,36]. In the dimer–dimer interphase, the side chain of L344 of a monomer from a dimer interacts with the respective L344‘s side chain of a monomer from the other dimer. When L344 is replaced by an Arg, the repulsion between the two positive charges should destabilise the tetramer. We engineered the introduction of a proline, L344P, into the α-helix to potentially disrupt the structure yielding, probably, an unfolded peptide [29,36]. 

As reported in the Section 2, 37TD-WT and 37TD-R337H were synthesised manually by SPPS (Solid Phase Peptide Synthesis) (Figure 1). These peptides were obtained using either a normal methionine or ^13^C-methyl methionine, in the latter case to facilitate NMR studies. All the other mutants were synthesised by microwave-assisted SPPS using ^13^C-methyl methionine (Figure 1). In this manuscript, we refer to the isotope-labelled sequences, unless stated otherwise. The data corresponding to the characterisation of the 37TDs are reported in the Supplementary Materials (Figures S1–S9 and Tables S1 and S2).

### 3.2. Secondary Structure of the 37TDs

**Figure 2:**
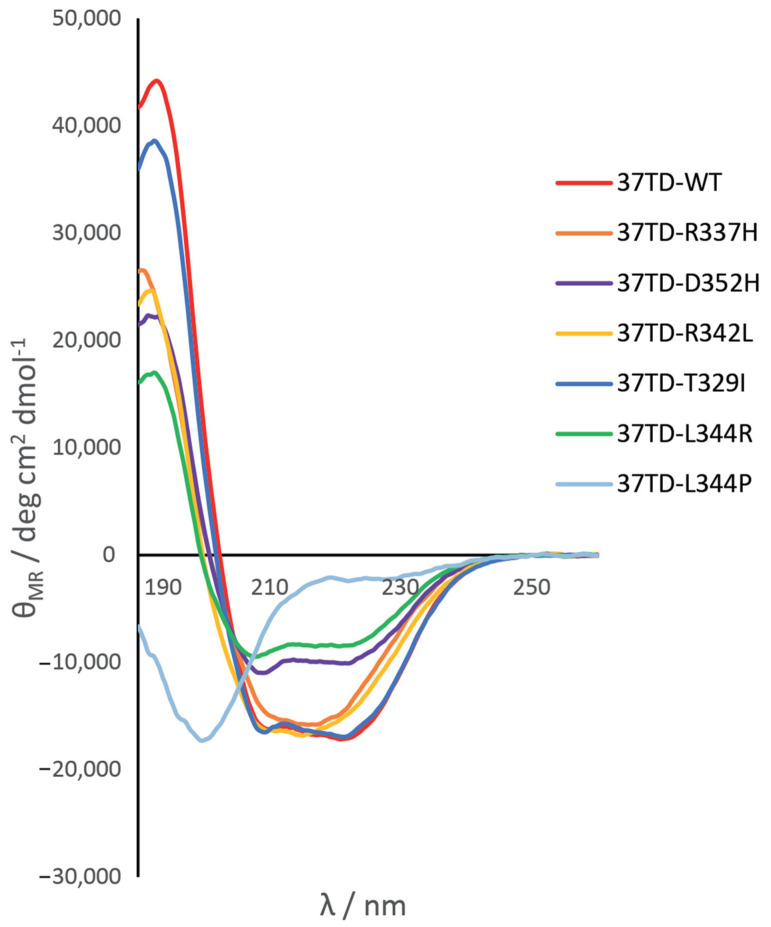
Circular dichroism spectra of the 37TDs. The spectra correspond to a monomer concentration of 20 μM of each peptide in 50 mM sodium phosphate buffer, pH 7. For 37TD-WT and 37TD-R337H, non-isotope-labelled peptides were used.

In order to obtain initial information on the conformation and folding of the synthetic tetramerisation domains (37TDs), we performed circular dichroism (CD) experiments. We studied our system in two distinct environments, namely, water and phosphate buffer, both at neutral pH, to check whether ionic strength influences the folding of the peptides. The spectra obtained are practically identical, implying that the peptides have the same structure under these conditions (Supplementary Materials, Figure S10). In CD experiments, non-isotope-labelled sequences of 37TD-WT and 37TD-R337H were used. 37TD-WT showed a typical profile of an α-helix with two negative bands at 208 and 222 nm (Figure 2) [61]. 37TD-T329I showed a very similar profile, characteristic of an α-helix, meaning that this peptide is also well folded and structured. Two peptides, 37TD-R337H and 37TD-R342L, presented a distinct profile where the contribution of the β-strain was more pronounced than in the other 37TDs. The band at 217 nm, typical of the β-strain, was more intense than the two bands of the α-helix, thus changing the profile of the spectra. 37TD-D352H and 37TD-L344R had similar profiles and, although both adopted an α-helix structure, they were less helical than the other 37TDs as the intensity of the bands was almost half the intensity of 37TD-WT. Finally, 37TD-L344P showed a random coil profile, indicating that the peptide was unstructured, as expected due to the introduction of a proline in the α-helix.

### 3.3. Determination of the Oligomeric State by Native MS

**Figure 3:**
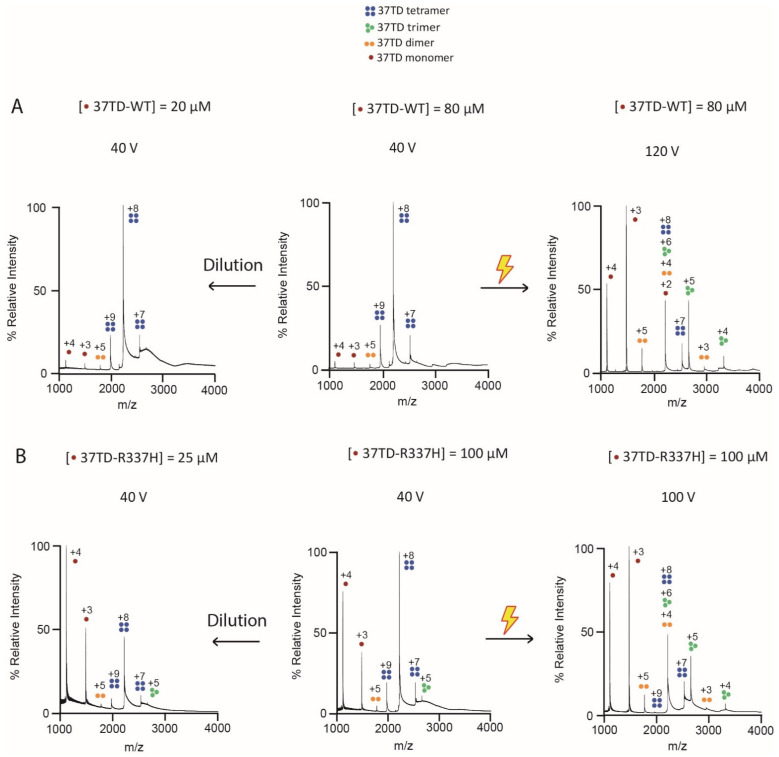
Native MS spectra of 37TD-WT (**A**) and 37TD-R337H (**B**). For both 37TDs, non-isotope-labelled peptides were used. The concentrations reported refer to the monomer. Starting from the central panel, which corresponds to the most native conditions of high concentration and low voltage, towards left is the spectrum of a more diluted sample, and towards right is a spectrum at higher voltage. The samples were dissolved in 200 mM ammonium acetate buffer, pH 7.

To determine the oligomeric state of the 37TDs, we performed native MS experiments. This technique allows the detection of non-covalent complexes using soft conditions of ionisation, such as electrospray ionisation (ESI) [62] and additional optimised instrumental parameters that preserve protein folding structures during the creation and ejection of ions from the solution to the gas phase. The experiments were performed using a time-of-flight (TOF) analyser, as well as Ion Mobility (IM) coupled to TOF. On the basis of different shapes and ions with the same *m*/*z* value, IM analysis allows an orthogonal measure to be added to the MS information and separates by drift times [45,63]. The peaks were assigned from both the MS spectra (Supplementary Materials, Table S3) and IM-MS analysis. Starting from the most native condition of 37TD-WT (80 µM, and low voltage, 40 V) (Figure 3A), we detected three consecutive charge states corresponding to the tetramer (+9, +8, +7). The contribution to different charge states was further validated by the ion mobilogram, which suggests that the most abundant species in the sample was the tetramer (Supplementary Materials, Figure S11). However, some signals of further oligomerisation were detected, such as octamers and decahexamers (16 monomers). Although the amount of these larger oligomers was low, as their peaks were not detected in the MS analysis, their presence may be due to aggregation of the peptide. To further test this hypothesis and to make sure that we were observing a specific oligomerisation event, we diluted the sample to 20 µM of monomeric 37TD-WT (Supplementary Materials, Figure S12). Under these conditions, peaks of the tetramer were still observed as the most intense, with a reduced contribution of larger oligomers. In the TOF spectrum (Figure 3A), low intensity peaks were assigned to both monomer (+4 and +3) and dimer (+5). Given the very low intensities of these peaks, we interpret them as products of sample ionisation. 

To study how the tetramer dissociates, we increased the cone voltage up to 80, 100 and 120 V. As expected, the tetramer was disrupted into smaller oligomers with increased activating conditions (Figure 3A and Supplementary Materials, Figure S11), and at 120 V the monomer was the most abundant species (+4, +3 and +2 charges). The tetramer, which was still present in both the MS and IM-MS spectra, dissociated mainly into monomers, as well as into trimers and dimers. As the p53TD is a dimer of dimers (Figure 1B), we initially expected to detect dimers as major species upon dissociation of the tetramer. However, the dissociation went directly towards the monomer. We interpret our results as an intrinsic instability of the dimer in this system. The dissociation constants are so low that they are at the limit of detection [5], making the dimer unstable and difficult to detect [36]. Kamada et al., although working with a slightly longer TD (Table 2), reported a mixture of tetramers and monomers for the WT in solution by gel filtration chromatography [29]. On the other hand, we consider the presence of the trimer in our experiments as a product of ionisation in the gas phase. Highly charged tetramers dissociated asymmetrically into trimers and monomers. This behaviour has been observed for other complexes where a unit is separated due to the high coulombic repulsion carrying a high charge and leaving the rest of the complex in a more stable form [64,65]. 

A different behaviour was observed for 37TD-R337H. In this case, at 100 µM and 40 V (Figure 3B and Supplementary Materials, Figure S13), the tetramer peaks were the most intense, but here, the monomer peaks were already more prominent. Similarly, as in the case of 37TD-WT, when the voltage was increased to 100 V, the tetramer dissociated mainly into monomers; however, peaks corresponding to trimers and dimers were also present. When the sample was diluted to 25 µM, the tetramer was still present, but the monomer was probably the most abundant species since its peaks were the most intense in the spectrum (Figure 3B and Supplementary Materials, Figure S14). These results confirm that the R337H mutation strongly destabilises the tetramer, as reported by others [55,56,57].

**Figure 4:**
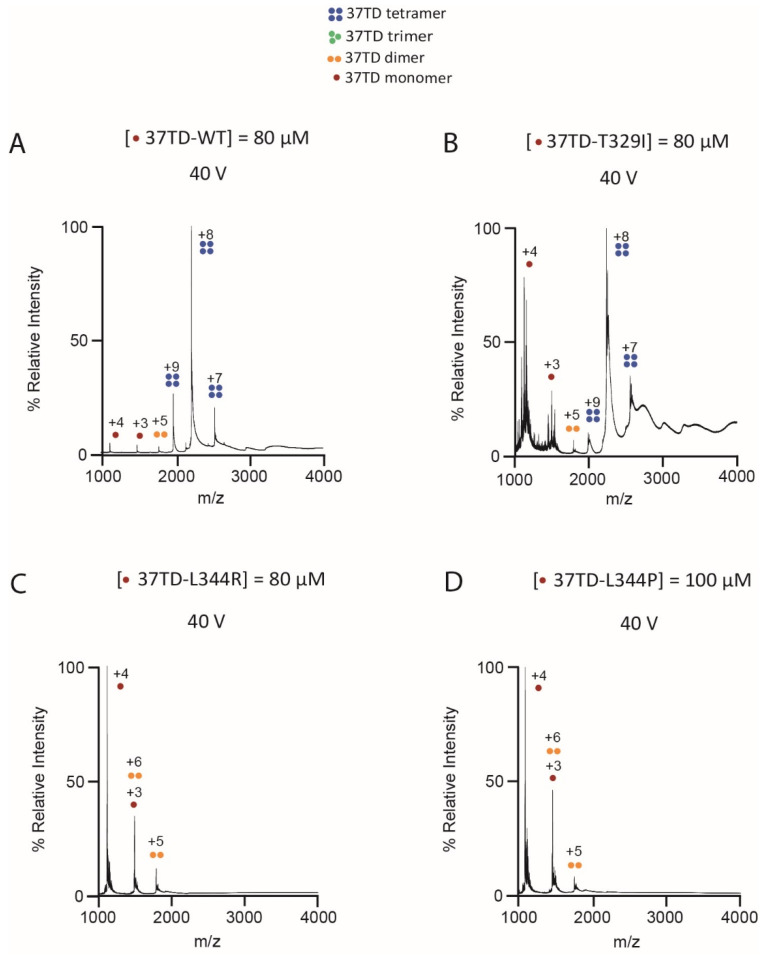
Native MS spectra of 37TD-WT (**A**), 37TD-T329I (**B**), 37TD-L344R (**C**) and 37TD-L344P (**D**). For 37TD-WT, a non-isotope-labelled sequence was used. The concentrations reported refer to the monomer. The samples were dissolved in 200 mM ammonium acetate buffer, pH 7. The tetramer-to-monomer ratio is compared in panels (**A**,**B**), whereas the dimer-to-monomer ratio is compared in panels (**C**,**D**).

A similar analysis was performed with the other 37TDs. We found that 37TD-T329I, 37TD-D352H and 37TD-R342L were mainly tetrameric, whereas 37TD-L344R and 37TD-L344P were mainly monomeric (Supplementary Materials, Figures S15–S24). Overall, the tetramer of all the 37TDs dissociated into smaller oligomers when the voltage was increased. The dissociation occurs similarly to 37TD-WT, forming mainly monomers accompanied by trimers and dimers. Concerning 37TD-T329I, at high concentration (80 µM) and low voltage (40 V), it was mainly tetrameric, although the monomer peaks were more intense compared to 37TD-WT (Figure 4A,B). By diluting the sample, the spectrum did not change (Supplementary Materials, Figures S19 and S20), and the tetramer dissociated with increased activating conditions. We conclude that this mutation does not stabilise the tetramer in the gas phase. 

Regarding the L344R and L344P mutations, we expected to detect the dimer and the monomer as the most abundant species, respectively. Looking at the TD as a dimer of dimers, the loss of the hydrophobic interaction between two L344 residues from two different dimers should promote the dissociation of the tetramer. In addition, the mutation L344R introduces a repulsion between two positively charged side chains. On the other hand, the introduction of a Pro in the α-helix, L344P, should destabilise the helix preventing peptide folding and oligomerisation. However, the corresponding MS spectra (Figure 4C,D) showed a mixture of monomers and dimers, where the monomer was the most abundant species. This means that the dimers of 37TD-L344R were not stable enough in the gas phase, and they dissociated into monomers despite applying low voltages and modified vacuums for ion cooling [66]. In the case of 37TD-L344P, although the proline disrupted the secondary structure of the peptide, resulting in an unfolded domain, as the CD results showed (Figure 2), some further oligomerisation was still possible. We conclude thus that these two mutants behave similarly in the gas phase.

Concerning 37TD-D352H and 37TD-R342L, both mutations strongly destabilised the tetramer, even more drastically than 37TD-R337H since the monomer was also the most abundant species at high concentration (Supplementary Materials, Figures S13–S18). D352 is the partner of R337 in the formation of the salt bridge in the WT. Mutations of either Arg337 or Asp352 have a strong destabilising effect because the salt bridge in the WT stabilises the dimer and, consequently, also the tetramer. In this study, either one residue or the other was replaced by His, and thus, the stability of the tetramer might depend on the protonation or deprotonation of the imidazole group. Finally, when Arg 342 was replaced by a Leu residue, the original intrahelical interactions of the Arg side chain with Glu residues (E339 and E346), which correspond to interactions *i, i − *3 and *i, i + *4, are lost. This loss may decrease helical stability, resulting in a less helical character in the CD profile (Figure 2) and, consequently, in a less stable tetramer.

### 3.4. Study of the Equilibrium between Oligomers by NMR

We conducted 2D ^1^H,^13^C-HSQC NMR experiments to monitor the ^13^C-methyl group of selectively ^13^C^ε^-Met340-labeled 37TDs. Each monomer contains only one Met and, upon oligomerisation, only one methyl signal was observed in the HSQC spectra due to the symmetry of the tetramer. Met340 is located in the tetramerisation interface, and the NMR signal of its methyl group is sensitive to the oligomerisation state of the TD, as reported by Bista et al. [12]. We analysed our 37TDs at a range of sample concentrations and temperatures in order to favour either small or large oligomers. For the mutations that introduce a His residue, we also examined how the stability of the tetramer was affected by a change in pH. 

**Figure 5:**
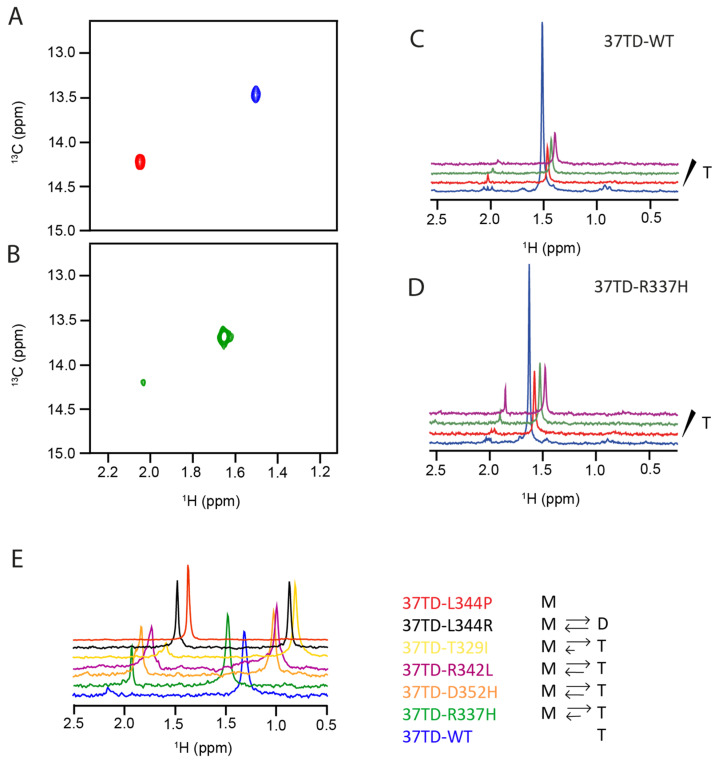
(**A**) 2D ^1^H,^13^C HSQC spectra at 25 °C of 37TD-WT (blue) and 37TD-L344P (red) at a monomer concentration of 20 μM. (**B**) 2D ^1^H,^13^C HSQC spectrum at 25 °C of 37TD-R337H (green) at a monomer concentration of 20 μM. ^1^H projections of ^1^H,^13^C HSQC spectra are represented in (**C**) for 37TD-WT and in (**D**) for 37TD-R337H. ^1^H projections of ^1^H,^13^C HSQC spectra of 100 μM 37TDs obtained at 25 °C (blue) and of 20 μM 37TDs acquired at 25 °C (red), 32 °C (green) and 40 °C (purple) (**C**,**D**). (**E**) ^1^H projections of HSQC spectra of 37TDs at 40 °C at a monomer concentration of 20 μM. A scheme illustrating the oligomeric 37TD species (T: tetramer, D: dimer, M: monomer) detected by NMR is shown on the **right** panel.

Starting from 37TD-WT, two concentrations (20 and 100 µM) were studied. In both cases, only one peak was detected in the HSQC spectra (Figure 5A,C). We expect the WT to be a tetrameric folded peptide, and indeed, the chemical shift ofthe cross-peak (^1^H 1.50 ppm, ^13^C 13.53 ppm) matches that previously reported for the tetramer (BMRB code 7251, [67]), [12]. We studied the WT system at different temperatures and pH (Supplementary Materials, Figure S25). These changes induced small chemical shift changes in the tetramer signal; however, in all the conditions studied, only one cross-peak was detected. We define this area of the spectrum as the region of folded peptides. 

Consistently with the CD and native MS results and with the fact that proline residues disrupt secondary structures, the NMR spectra of 37TD-L344P were found to be typical of unfolded peptides, and they show very low signal dispersion. This finding was clearly seen in the methyl region of the 1D ^1^H spectra (0.8–0.9 ppm). The methyl signals of residues Ile332, Leu344 and Leu330, located in the hydrophobic core of the tetramer, were shifted upfield in the 1D ^1^H spectra of 37TD-WT (Ile332 δH^δ^ = 0.78 ppm, Leu344 δH^δ^ = 0.74 ppm, Leu330 δH^δ^ = 0.54 ppm) (Supplementary Materials, Figure S32). In contrast, in the unstructured mutant 37TD-L344P, where the hydrophobic tetrameric interface is lost, all the methyl groups virtually completely overlapped and resonated at ca. 0.9 ppm. The ^1^H,^15^N HSQC spectrum of 37TD-L344P, obtained at natural abundance, showed a similar lack of signal dispersion for the amide NH resonances (Supplementary Materials, Figure S33), thereby further confirming the unstructured nature of this peptide. In the ^1^H,^13^C-HSQC spectra of 37TD-L344P (Figure 5A), a single cross-peak resonating at 2.05 ppm was detected, close to the expected value (2.10 ppm) for a random coil peptide [68]. This observation allows us to define the region where the signal of an unfolded 37TD peptide appears.

To assign the oligomeric state of the species present in solution, we performed X-STE NMR diffusion experiments. These experiments measure translational diffusion coefficients (D) of molecules containing ^1^H nuclei attached to magnetically active heteronuclei [48]. On the base of the D values obtained by diffusion NMR, it is possible to estimate the hydrodynamic radius (R_H_) of the species analysed. The diffusion coefficient determined for 37TD-WT was D = (1.00 ± 0.02) × 10^−10^ m^2^ s^−1^ providing an R_H_ of 22.2 Å, close to the expected value (20.3 Å) for a globular protein of the size of tetrameric 37TD (148 residues) (Table 3 and Supplementary Materials, Figure S34) [50]. For 37TD-L344P, the values obtained were D = (1.45 ± 0.01) × 10^−10^ m^2^ s^−1^ and R_H_ = 15.3 Å, in agreement with the R_H_ predicted (14.6 Å) for a disordered 37-residue peptide (Table 3 and Supplementary Materials, Figure S35) [50]. We conclude that 37TD-WT is a folded tetramer, and 37TD-L344P is an unfolded monomer.

In the ^1^H,^13^C-HSQC spectra of 37TD-L344P, when the temperature was decreased to 5 °C, a second small peak appeared (^1^H 1.74 ppm, ^13^C 13.99 ppm) in a region between the signal of the folded 37TD-WT peptide and the signal of the unfolded 37TD-L344P peptide (Supplementary Materials, Figure S31). We thus assign this peak to a larger oligomer, most probably a dimer since in MS experiments, some signals of the dimer were detected. Our results suggest that although this peptide is unstructured, some oligomerisation occurs in the gas phase or at low temperature. 

Having defined the regions of folded and unfolded peptides, we analysed the different oligomeric species detected in the ^1^H,^13^C-HSQC spectra of the other 37TDs assigning their oligomeric state based on the MS results and previous data reported in the literature. Upon 37TD mutation, if the domain remains folded, the methyl cross-peak may, to some extent, be shifted with respect to that of the tetrameric WT due to differences in the chemical environment of Met340. For example, the ^1^H chemical shift in the methyl group of Met340 may be significantly affected by changes in the orientation of the aromatic ring of Phe341 since both side chains are in close proximity in the three-dimensional structure of tetrameric p53TD (PDB ID 1OLG) [69]. On the other hand, on unfolded peptides, due to conformational averaging, the methyl signal should appear close to the expected random coil value (2.10 ppm) and should not vary much between mutants. 

In the case of 37TD-R337H, a single peak that resonated at the position close to that of the tetrameric WT (^1^H 1.64 ppm, ^13^C 13.67 ppm) was detected at high concentration (100 µM), and an additional weak cross-peak (^1^H 2.02 ppm, ^13^C 14.19 ppm) appeared upon dilution of the sample (20 µM) (Figure 5B,D). By increasing the temperature and/or the pH, the relative intensity of the smaller cross-peak increased (Supplementary Materials, Figure S26). Consistently with the disruption of tetramer formation by increasing the temperature and the destabilisation of the tetramer by deprotonation of the imidazole group [55], we assign the main cross-peak signal to the tetramer and the weak cross-peak signal to a smaller oligomer. A comparison of the ^1^H,^13^C-HSQC spectra (Figure 5A,B) revealed that the weak 37TD-R337H cross-peak was very close to the signal of 37TD-L344P. Together with the MS results (Figure 3B), where a mixture of tetramer and monomer was detected, we assign this peak to the 37TD-R337H monomer. Therefore, our NMR experiments confirm that the R337H mutation strongly destabilises the tetramer, thus complementing our previous native MS results. Our findings using both techniques suggest that the disruption of the tetramer proceeds towards the monomer, whereas the dimer is probably unstable, or its concentration is below the limit of detection.

The T329I mutation is reported to have a stabilising effect on the tetramer [29]. The HSQC of 37TD-T329I showed a major peak that overlaps with the signal of 37TD-WT, indicating that the peptide was mainly tetrameric, as expected. Nevertheless, a small peak in the unfolded region was also present, even at high concentration (Supplementary Materials, Figure S29). We conclude that this domain is mainly a structured and folded peptide, but it coexists with a small population of unstructured peptide, and therefore, it does not stabilise the tetramer in the gas phase or in solution. 

The HSQC of 37TD-D352H showed a mixture of tetramer and monomer even at high peptide concentration (Supplementary Materials, Figure S27), indicating that this mutant destabilises the tetramer. When the temperature was increased, the peak corresponding to the folded 37TD-D352H species was significantly shifted downfield. However, protonation of the imidazole group did not seem to affect the oligomeric state of 37TD-D352H, and the HSQC spectra recorded at pH 7 and 5 were almost identical.

Regarding the R342L mutation, the HSQC spectra of 37TD-R342L showed a mixture of monomer and tetramer, where the relative peaks had a similar intensity. By changing the concentration and the temperature, 37TD-R342L behaved in a similar manner to 37TD-D352H (Figure 5E and Supplementary Materials, Figure S28).

Finally, the HSQC spectra of 37TD-L344R showed two peaks (Figure 5E and Supplementary Materials, Figure S30). One (^1^H 2.04 ppm, ^13^C 14.24 ppm) corresponds to the unfolded monomeric peptide, whereas the other (^1^H 1.58 ppm, ^13^C 13.52 ppm) resembles that of the folded tetrameric species. However, our MS experiments showed a mixture of monomer and dimer for this peptide, in agreement with a previous study that reported that this mutant was mainly dimeric [29]. To further analyse the size of the oligomeric species present in solution, we performed X-STE NMR diffusion experiments, which allowed us to independently monitor the molecular diffusion of each 37TD-L344R species present in solution (Table 3 and Supplementary Materials, Figure S36).

For the monomeric 37TD-L344R species, we obtained D = (1.78 ± 0.04) × 10^−10^ m^2^ s^−1^ providing an R_H_ of 12.5 Å, a value smaller than that predicted for a disordered 37-residue peptide (14.6 Å) (Table 3), suggesting that the monomeric 37TD-L344R peptide has some degree of compaction. For the 37TD-L344R species resonating at δ^1^H = 1.58 ppm and δ^13^C = 13.52 ppm, we determined a D = (1.38 ± 0.02) × 10^−10^ m^2^ s^−1^, which provided an R_H_ value of 16.1 Å, in good agreement with the value predicted (16.5 Å) for a globular protein of the size of dimeric 37TD (74 residues) (Table 3). These results indicate that monomeric and dimeric 37TD-L344R coexist in solution.

A qualitative scheme illustrating the equilibrium between different species of the 37TDs based on NMR data at a monomer concentration of 20 μM and 40 °C, is represented in Figure 5E.

## 4. Conclusions

In our study, we applied native MS, which received increased interest and application in recent years, because it is a powerful technique to detect the native state of complexes, and it allows us to conduct the structural analysis of protein or peptide complexes while intact. Additionally, those complexes were analysed and characterised by NMR, a potent and informative technique in terms of structural and conformational analysis in solution. From our experiments, we conclude that all the mutations studied destabilised the tetramer, whereas the monomer population significantly increased. Those results suggest that the isolated dimer may be unstable and practically absent in the oligomer equilibria. Due to the increasing amount of data supporting the idea that p53 dimer is the prevalent form of the wild-type protein in the cytosol [5,7,36], we believe that our results should be taken into account in future biological studies about the oncogenic mechanisms associated with the mutated forms of p53. To be more precise, abolishing all those protein–protein interactions where p53 is involved could be a crucial component of the oncogenic behaviour of cancer-associated p53 TD mutants. 

## Figures and Tables

**Table 1:** Conditions of native MS experiments.

Source T	Bias	Trap	Transfer	Backing	Wave Velocity (IM)	Wave Height (IM)
40 °C	4 V (TOF)	6 V	4 V	5.7–5.9 mbar	300 m/s	8 V
	15 V (IM)					

**Table 2:** Definition of the TDs.

Residues	Reference
325–353	Bista et al. [12]
325–355	Rajagopalan et al. [5]
326–356	Gaglia et al. [7]
323–360	Tidow et al. [51]
320–356	García [52], Gordo [53]
319–358	Kamada et al. [29]

**Table 3:** Diffusion coefficients (D) and hydrodynamic radius (R_H_) of 37TD-WT, 37TD-L344P, and 37TD-L344R. For 37TD-L344R, the two species are independently analysed. Predicted R_H_ are calculated from [50].

37TDs	D × 10^−10^/m^2^ s^−1^	R_H_/Å Experimental	R_H_/Å Predicted
37TD-WT	1.00 ± 0.02	22.2	20.3 ^a^
37TD-L344P	1.45 ± 0.01	15.3	14.6 ^b^
37TD-L344R Dimer Monomer			
	1.38 ± 0.02	16.1	16.5 ^c^
	1.78 ± 0.04	12.5	14.1 ^b^
		

^a^ Value calculated for a folded tetramer. ^b^ Value calculated for a disordered monomer. ^c^ Value calculated for a folded dimer.

## Data Availability

Data sharing is not applicable.

## Supplementary Materials

The following supporting information can be downloaded at: https://www.mdpi.com/article/10.3390/cimb45060317/s1.
