# Supplementary material for: How Do Cancer-Related Mutations Affect the Oligomerisation State of the p53 Tetramerisation Domain?
Source: Curr Issues Mol Biol. 2023 Jun 7;45(6):4985–5004. doi: 10.3390/cimb45060317 (PMC10296842; doi:10.3390/cimb45060317)
Supplement: Supplementary file 1 [file cimb-45-00317-s001.zip › cimb-2420268-supplementary.pdf]

Supplementary Material

How do cancer-related mutations affect the oligomerisation state of the p53 tetramerisation domain?

Federica Nicolini[1], Toni Todorovski[1,2,§], Eduard Puig[1], Mireia Diaz-Lobo[1], Marta Vilaseca[1], Jesús García[1], David Andreu[2], Ernest Giralt[1,3,*]

[1] Institute for Research in Biomedicine (IRB Barcelona), Baldiri Reixac 10, 08028 Barcelona, Spain

[2] Department of Experimental and Health Sciences, Universitat Pompeu Fabra, Barcelona Biomedical Research Park, Dr. Aiguader 88, 08003 Barcelona, Spain

[§] Current affiliation: University of Rijeka, Department of Biotechnology, 51000 Rijeka, Croatia

[3] Department of Inorganic and Organic Chemistry, University of Barcelona, Martí i Franquès, 1-11, 08028 Barcelona, Spain

37TDs	Molecular formula	Calc. MW	Found MW[a]	t_R UPLC or HPLC min	% Purity[b]
**37TD-WT (not labelled)**	$C_{197} H_{312} O_{59} N_{56} S$	4438,28559	4438,28981	16,722	> 95
**37TD-WT**	$C_{196} {}^{13}C H_{312} O_{59} N_{56} S$	4439,28894	4439,29224	16,881	> 95
**37TD-R337H (not labelled)**	$C_{197} H_{307} O_{59} N_{55} S$	4419,24339	4419,24725	16,625	> 95
**37TD-R337H**	$C_{196} {}^{13}C H_{307} O_{59} N_{55} S$	4420,24674	4420,25107	16,656	> 95
**37TD-D352H**	$C_{198} {}^{13}C H_{314} O_{57} N_{58} S$	4461,32091	4461,32512	9,210	> 95
**37TD-R342L**	$C_{196} {}^{13}C H_{311} O_{59} N_{53} S$	4396,27189	4396,27627	10,036	85
**37TD-T329I**	$C_{198} {}^{13}C H_{316} O_{58} N_{56} S$	4451,32533	4451,33161	9,770	90
**37TD-L344R**	$C_{196} {}^{13}C H_{313} O_{59} N_{59} S$	4482,30599	4482,31032	8,489	> 95
**37TD-L344P**	$C_{195} {}^{13}C H_{308} O_{59} N_{56} S$	4423,25764	4423,25888	8,634	86

Table S1. Molecular weight, retention times and purity of the synthetic peptides. [a]Measured by LQT-FT MS. [b]Measured by UPLC for 37TD-WT and 37TD-R337H sequences (either labelled or not) and by HPLC for the other 37TDs. A gradient of 0 − 70 % acetonitrile in water in 40 min was applied in the UPLC while a gradient of 5 – 60 % acetonitrile in water in 15 min was used in the HPLC.

		**37TDs**							
		37TD-WT (not labelled)	37TD-WT	37TD-R337H (not labelled)	37TD-R337H	37TD-D352H	37TD-R342L	37TD-T329I	37TD-L344R
**Charges**	+1	4442,01	4443,01	4422,96	4423,96	4465,08	4399,99	4455,08	4486,05
	+2	2221,51	2222,00	2211,98	2212,48	2233,04	2200,50	2228,04	2243,53
	+3	1481,34	1481,67	1474,99	1475,32	1489,02	1467,33	1485,69	1496,02
	+4	1111,25	1111,50	1106,49	1106,74	1117,02	1100,75	1114,52	1122,26
	+5	889,20	889,40	885,39	885,59	893,82	880,80	891,82	898,01
	+6	741,17	741,34	737,99	738,16	745,01	734,16	743,35	748,51
	+7	635,43	635,57	632,71	632,85	638,72	629,43	637,30	641,72
	+8	556,13	556,25	553,74	553,87	559,01	550,87	557,76	561,63
		37TD-L344P							
**Charges**	+1	4426,98							
	+2	2213,99							
	+3	1476,33							
	+4	1107,50							
	+5	886,20							
	+6	738,66							
	+7	633,28							
	+8	554,25							

Table S2. Masses over charges (m/z) of the 37TDs.

37TD-WT: Ac-KKPLDGEYFTLQIRGRERFEMFRELNEALELKDAQAG-NH_2

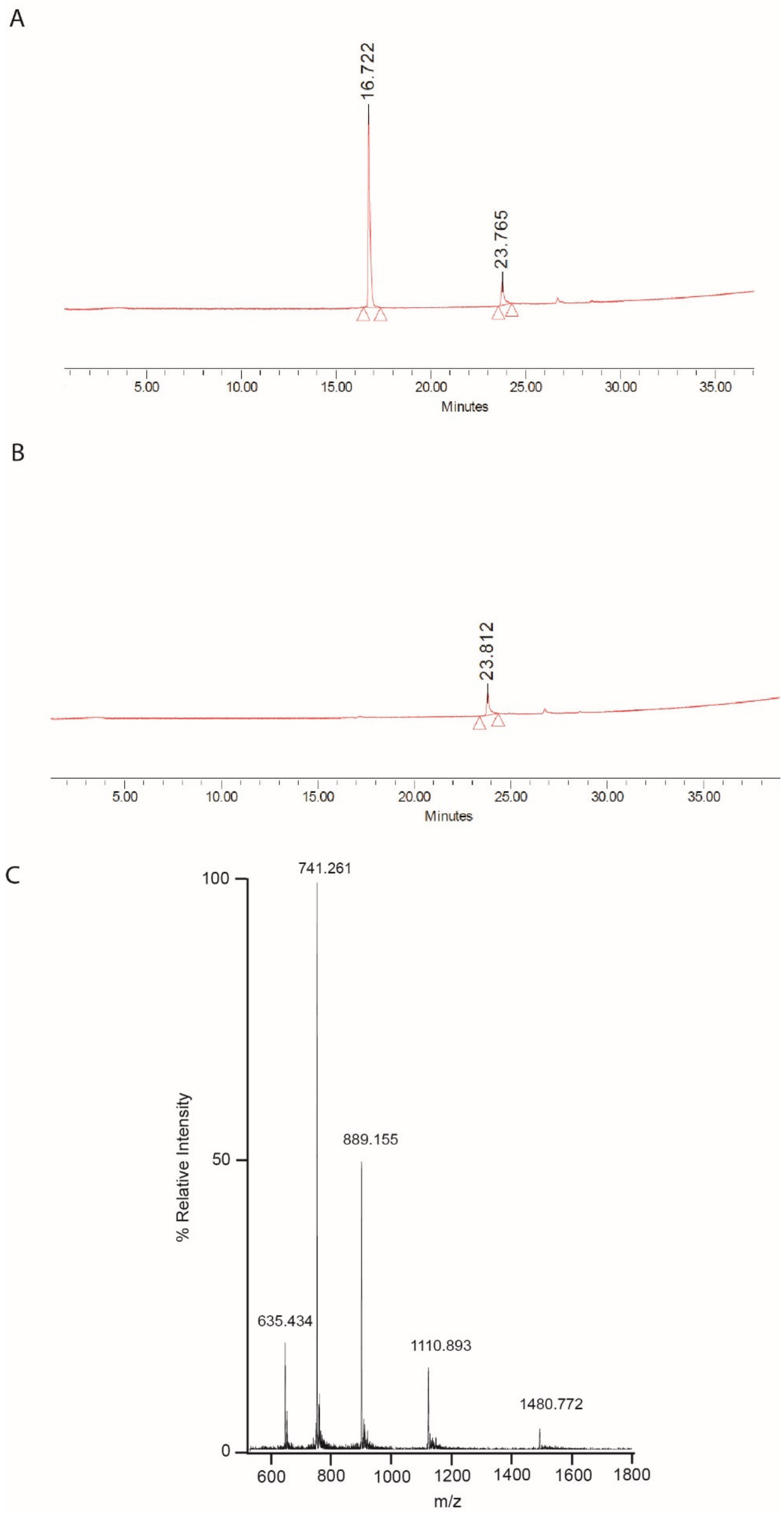

Figure S1. UPLC (A, B) and UPLC-MS (C) of unlabelled 37TD-WT (A and C) and a blank run (B). Gradient of 0 – 70 % acetonitrile in water in 40 min.

37TD-WT: Ac-KKPLDGEYFTLQIRGRERFEMFRELNEALELKDAQAG-NH_2

M ^{13}C-methyl methionine

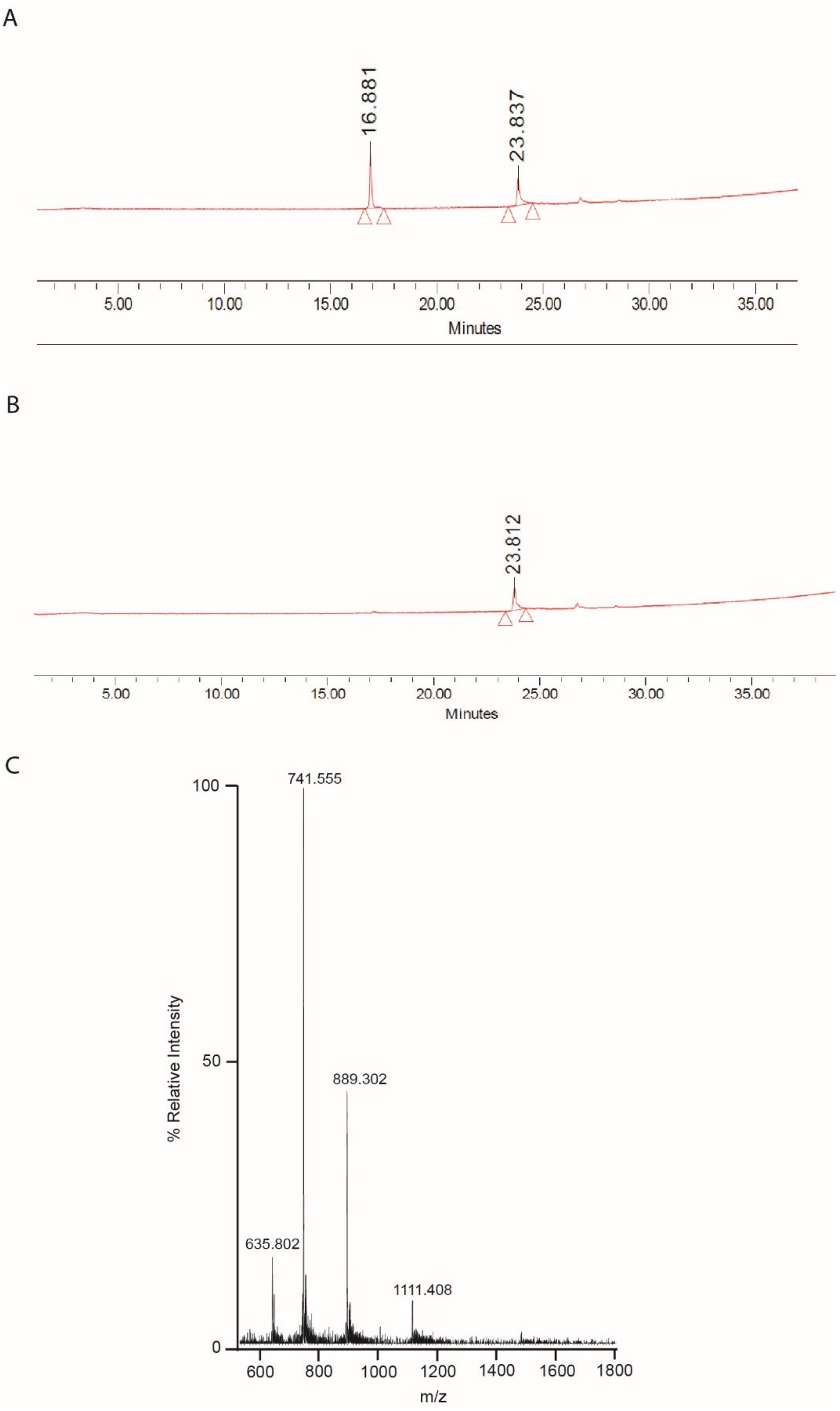

Figure S2. UPLC (A, B) and UPLC-MS (C) of 37TD-WT (A and C) and a blank run (B). Gradient of 0 – 70 % acetonitrile in water in 40 min.

37TD-R337H: Ac-KKPLDGEYFTLQIRGREHFEMFRELNEALELKDAQAG-NH_2

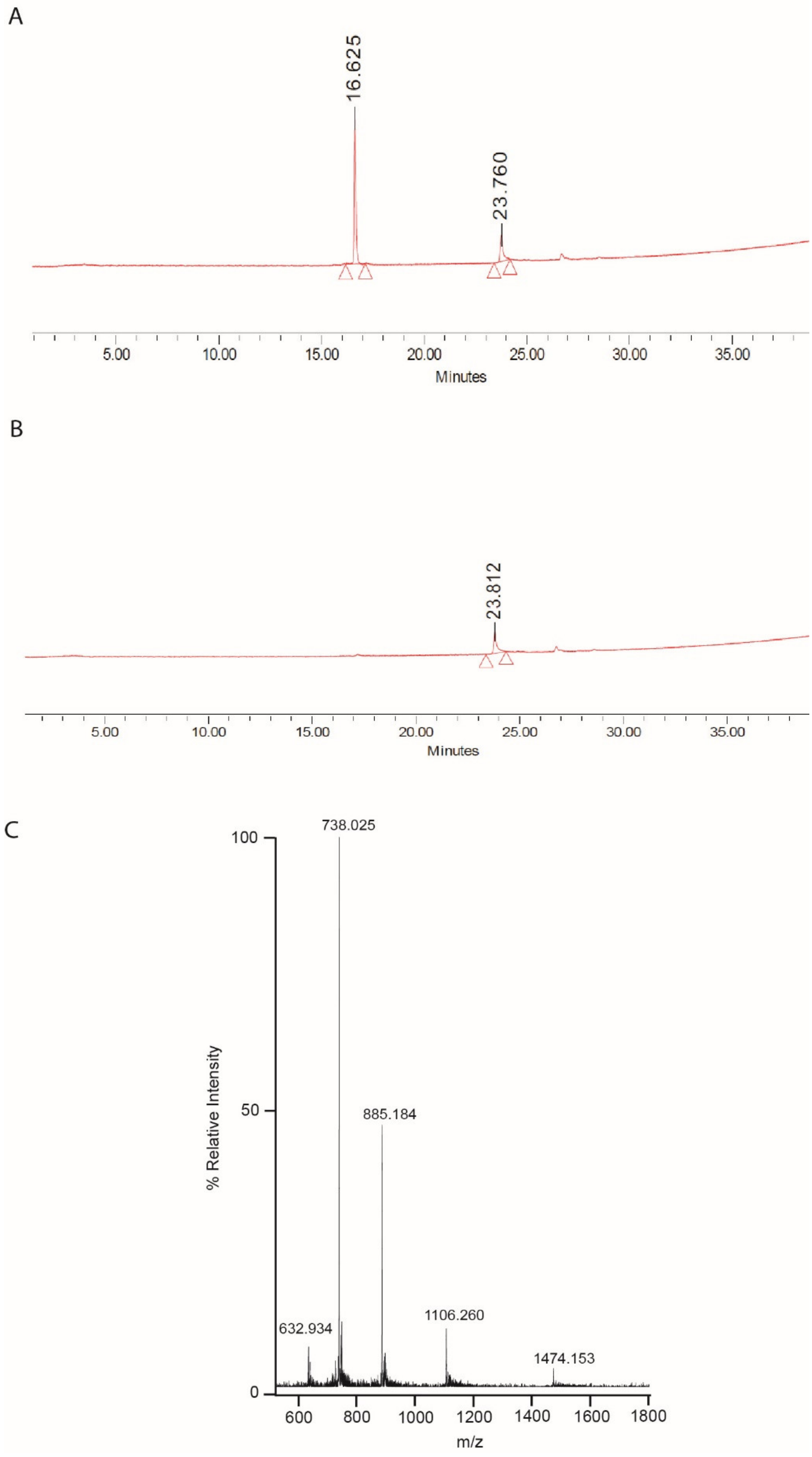

Figure S3. UPLC (A, B) and UPLC-MS (C) of unlabelled 37TD-R337H (A and C) and a blank run (B). Gradient of 0 – 70 % acetonitrile in water in 40 min.

37TD-R337H: Ac-KKPLDGEYFTLQIRGREHFEMFRELNEALELKDAQAG-NH_2

M ^{13}C-methyl methionine

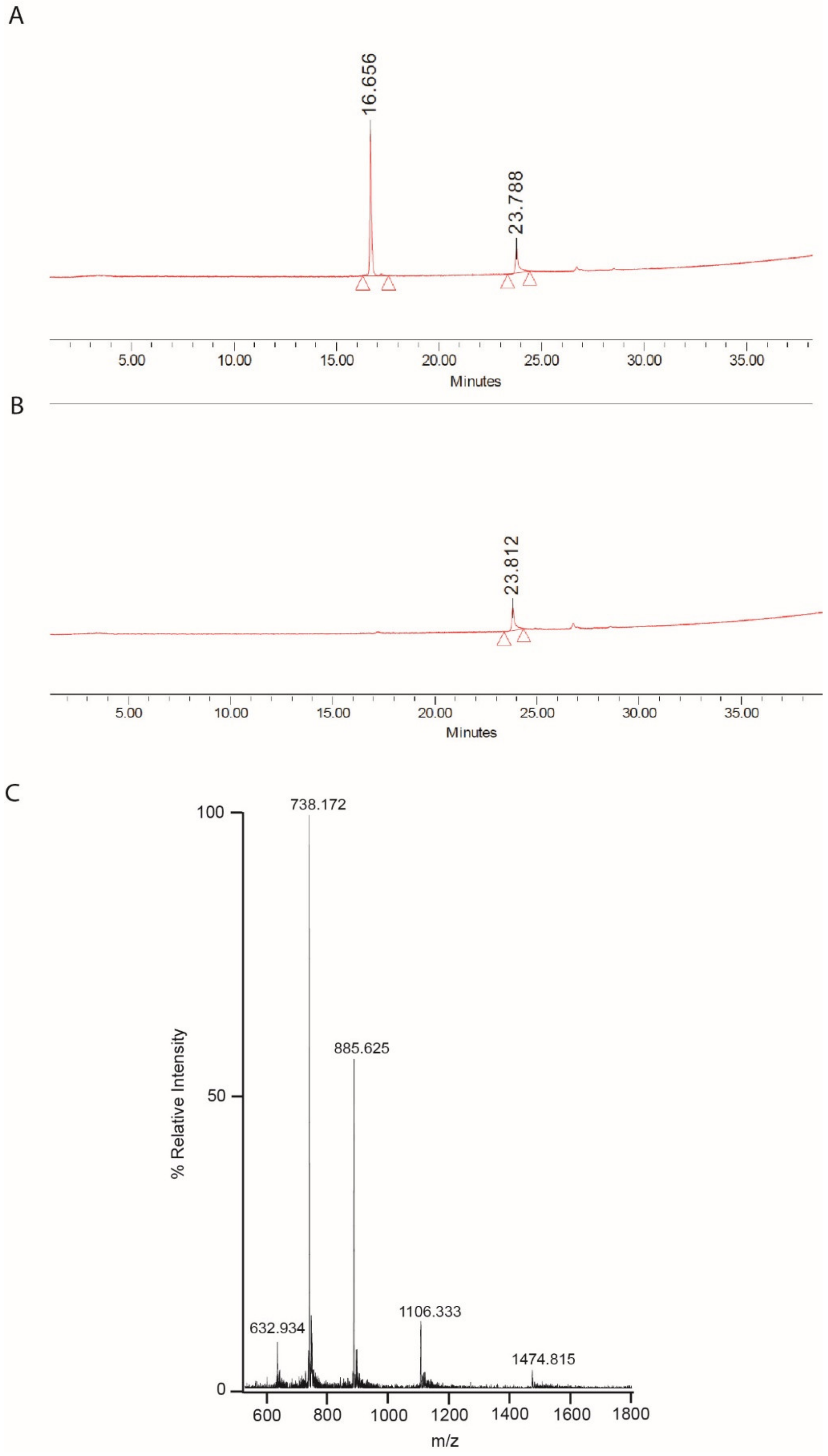

Figure S4. UPLC (A, B) and UPLC-MS (C) of 37TD-R337H (A and C) and a blank run (B). Gradient of 0 – 70 % acetonitrile in water in 40 min.

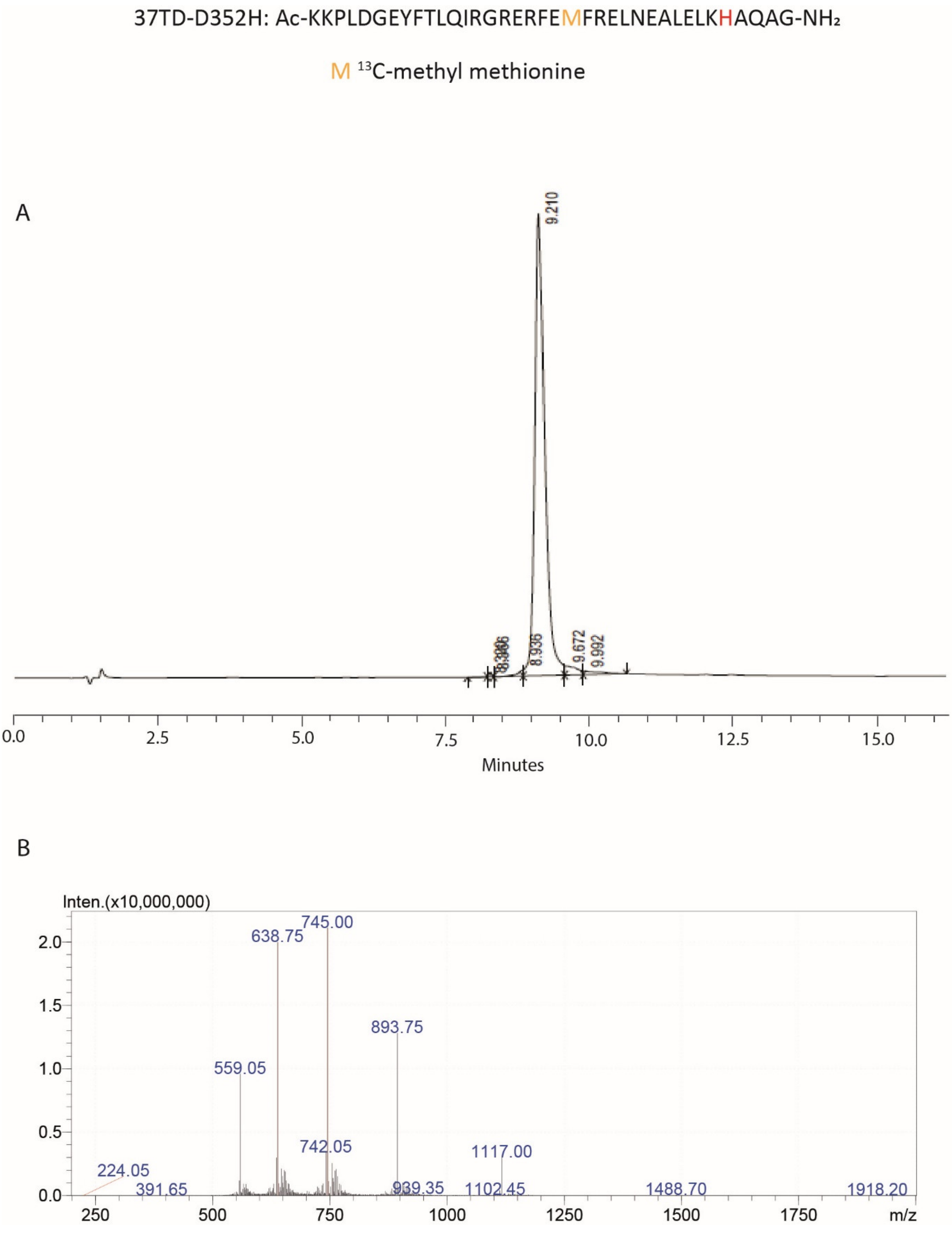

Figure S5. HPLC (A) and HPLC-MS (B) of 37TD-D352H. Gradient of 5 – 60 % acetonitrile in water in 15 min.

37TD-R342L: Ac-KKPLDGEYFTLQIRGRERFEMFLELNEALELKDAQAG-NH_2

M ^{13}C-methyl methionine

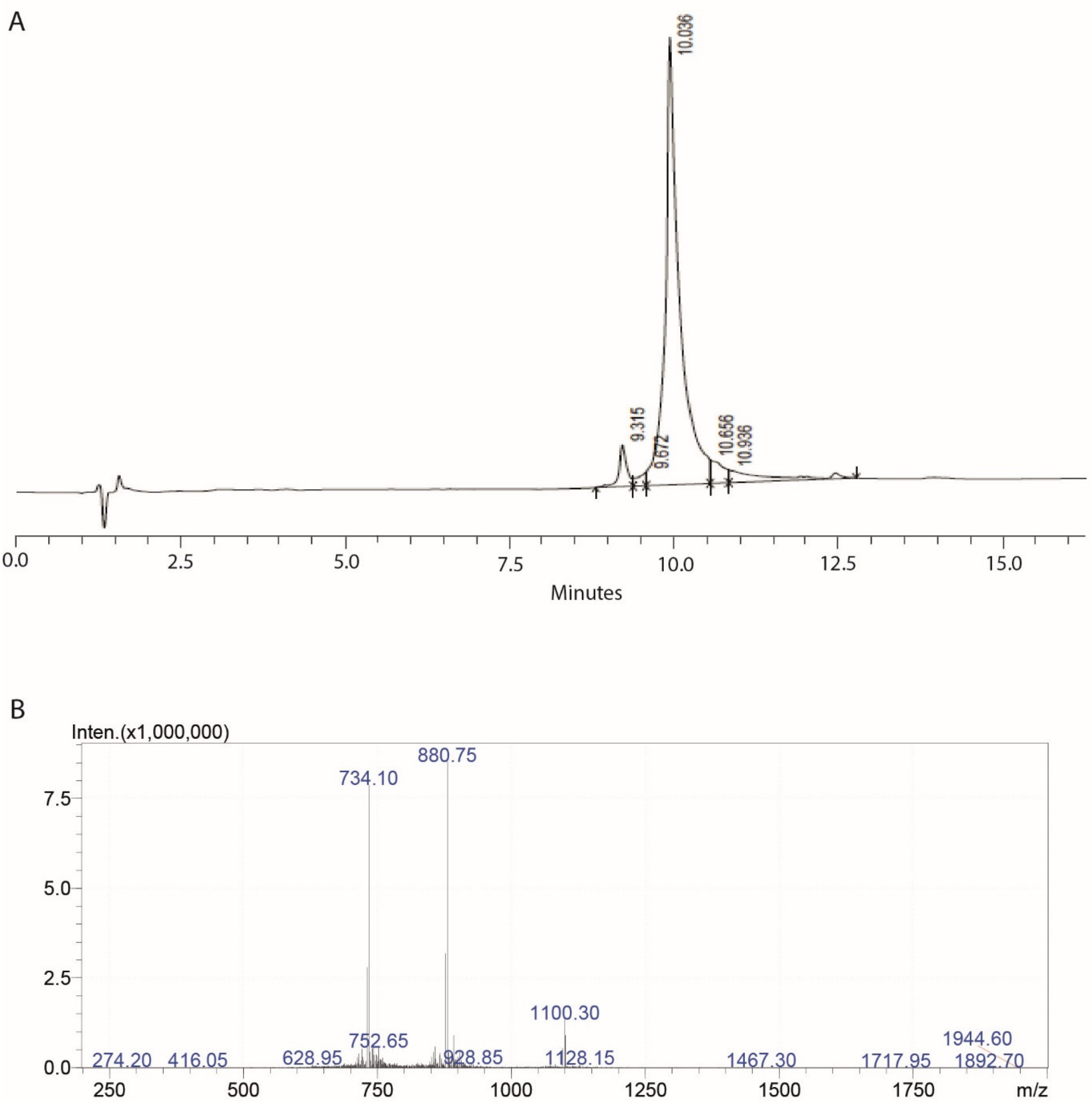

Figure S6. HPLC (A) and HPLC-MS (B) of 37TD-R342L. Gradient of 5 – 60 % acetonitrile in water in 15 min.

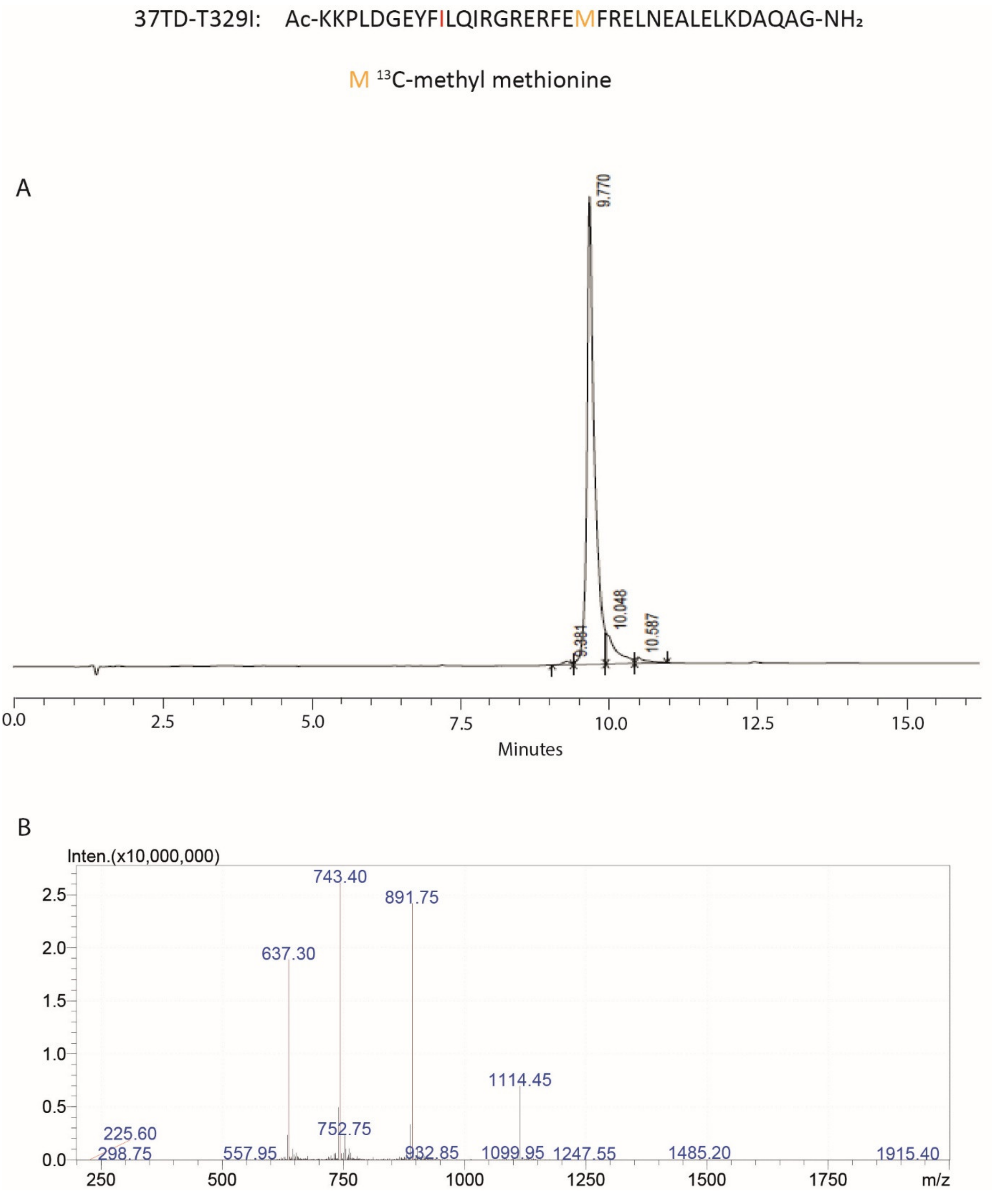

Figure S7. HPLC (A) and HPLC-MS (B) of 37TD-T329I. Gradient of 5 – 60 % acetonitrile in water in 15 min.

37TD-L344R: Ac-KKPLDGEYFTLQIRGRERFEMFRERNEALELKDAQAG-NH_2

M ^{13}C-methyl methionine

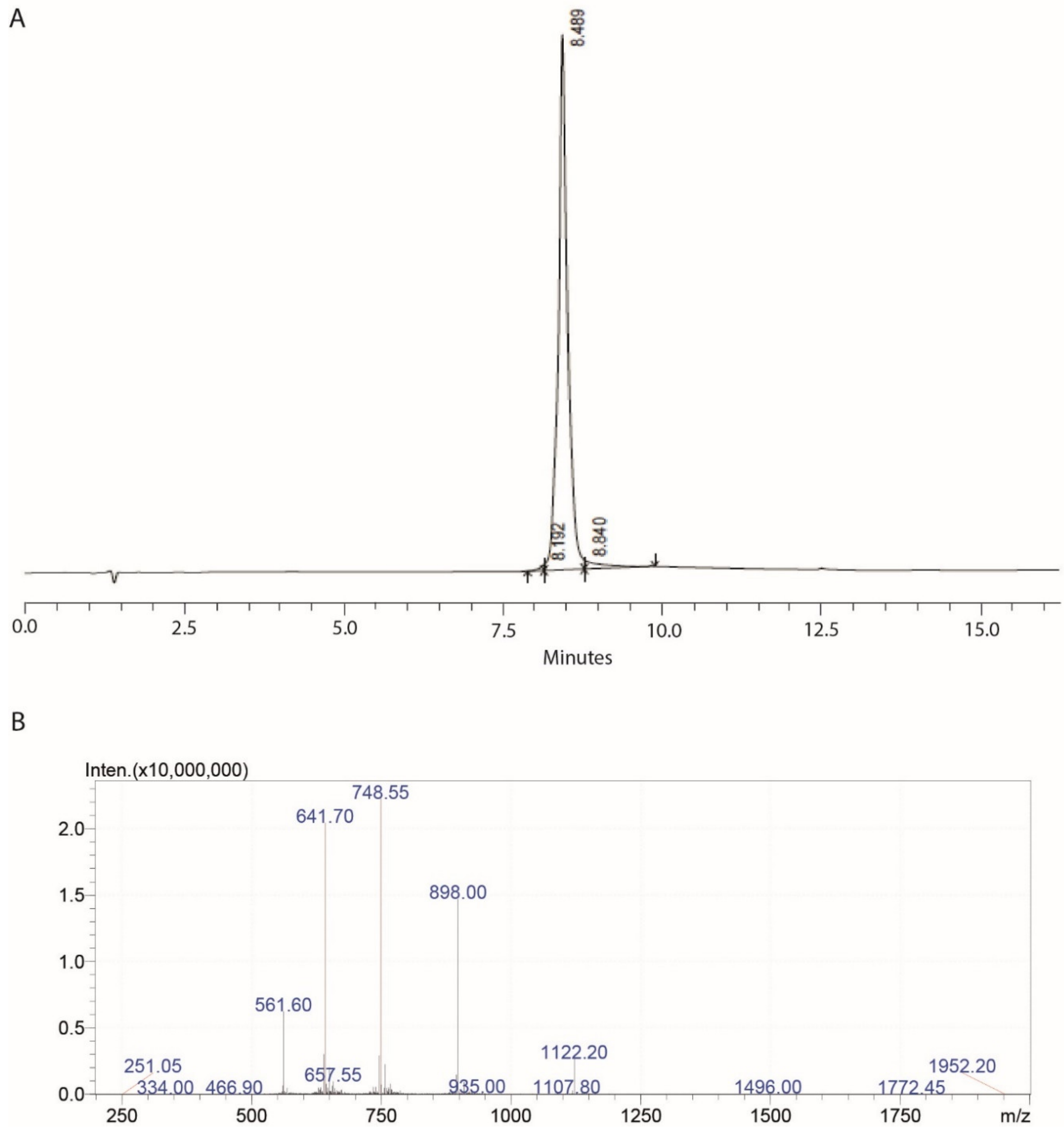

Figure S8. HPLC (A) and HPLC-MS (B) of 37TD-L344R. Gradient of 5 – 60 % acetonitrile in water in 15 min.

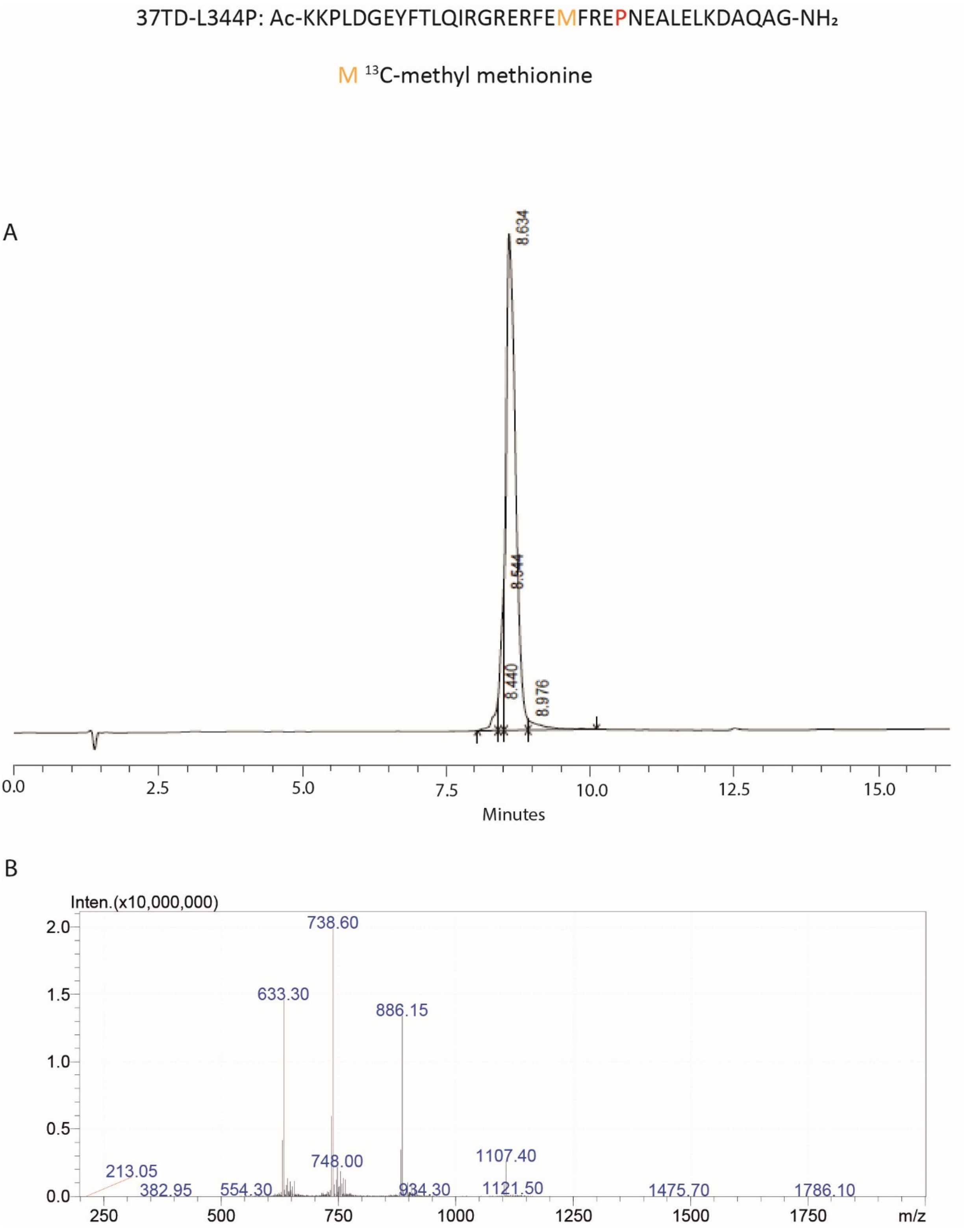

Figure S9. HPLC (A) and HPLC-MS (B) of 37TD-L344P. Gradient of 5 – 60 % acetonitrile in water in 15 min.

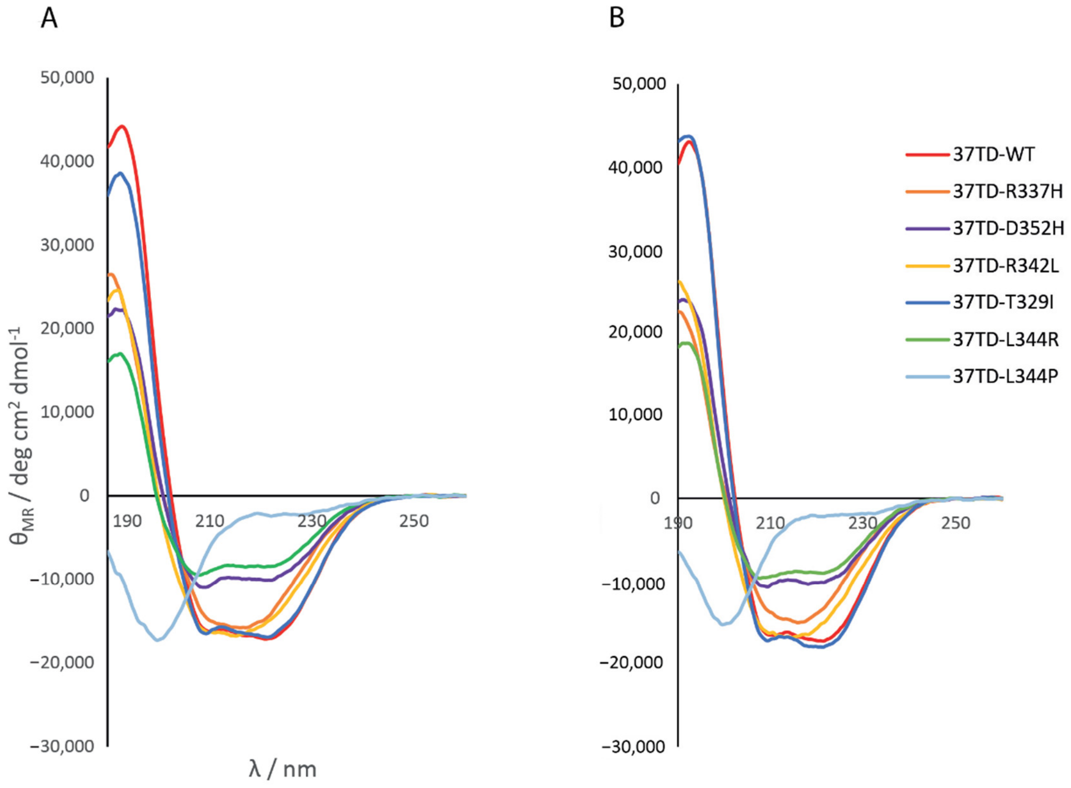

Figure S10. Circular dichroism spectra of the 37TDs in 50 mM sodium phosphate buffer, pH 7 (A) and water, pH 7 (B). The spectra correspond to a monomer concentration of 20 µM of each peptide. For 37TD-WT and 37TD-R337H, non isotope-labelled peptides were used.

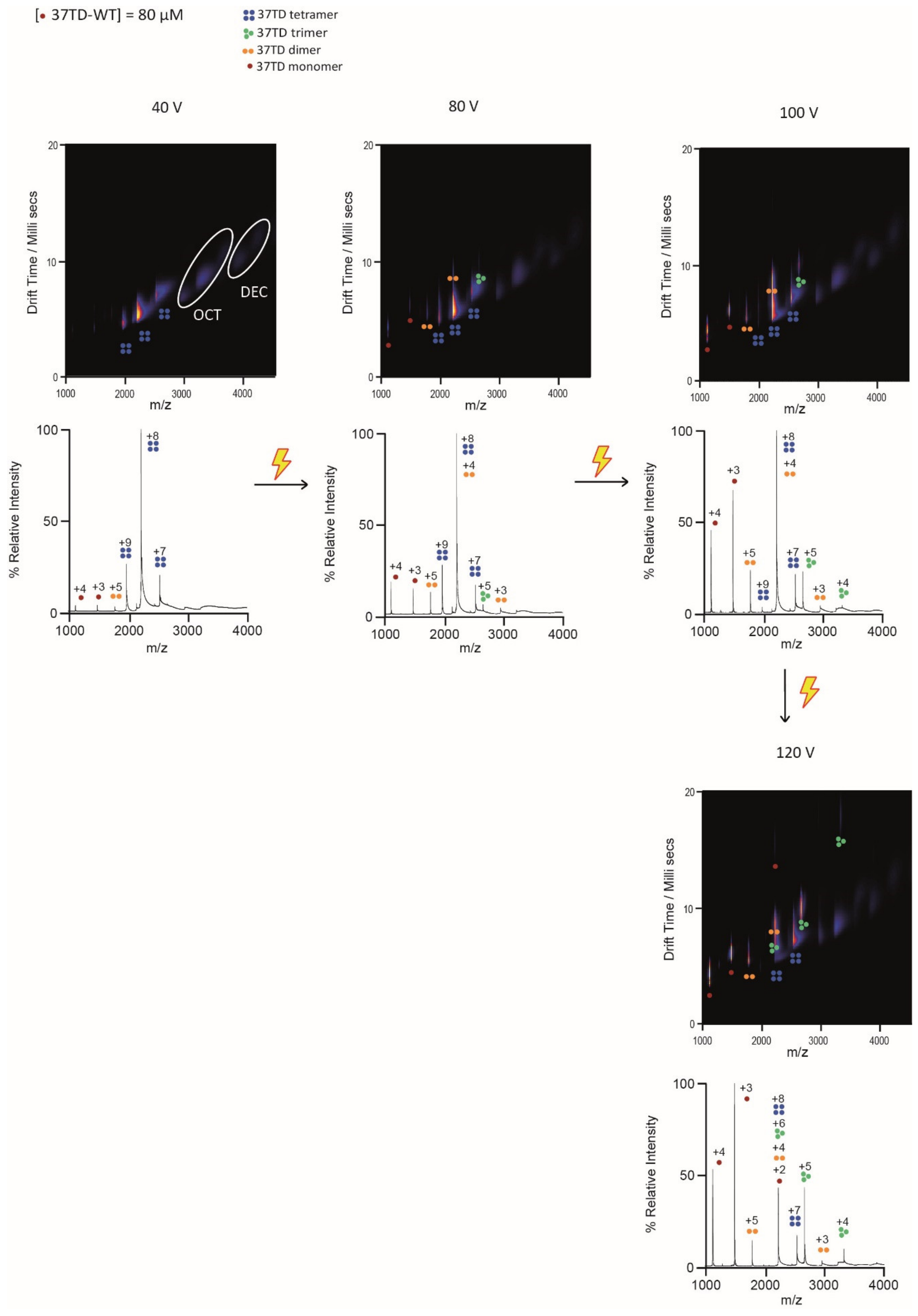

Figure S11. Native Mass Spectrometry and Ion Mobility spectra of 80 µM 37TD-WT at different cone voltages (40, 80, 100, and 120 V). Non isotope-labelled peptide was used. The concentrations reported

always refer to the monomer. The sample was dissolved in 200 mM ammonium acetate buffer, pH 7. OCT: octamer; DEC: decahexamer (16 monomers).

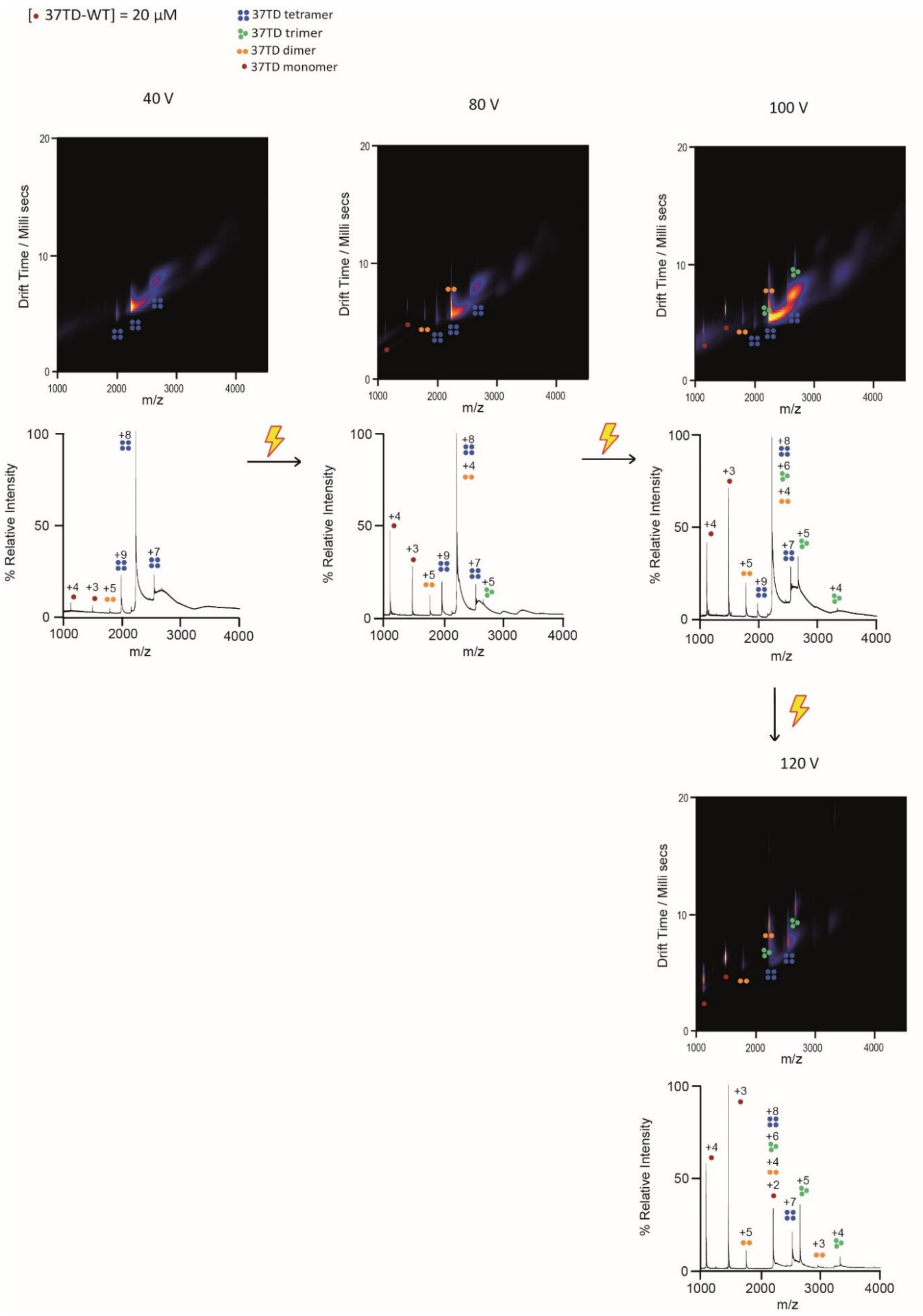

Figure S12. Native Mass Spectrometry and Ion Mobility spectra of 20 μM 37TD-WT at different cone voltages (40, 80, 100, and 120 V). Non isotope-labelled peptide was used. The concentrations reported always refer to the monomer. The sample was dissolved in 200 mM ammonium acetate buffer, pH 7.

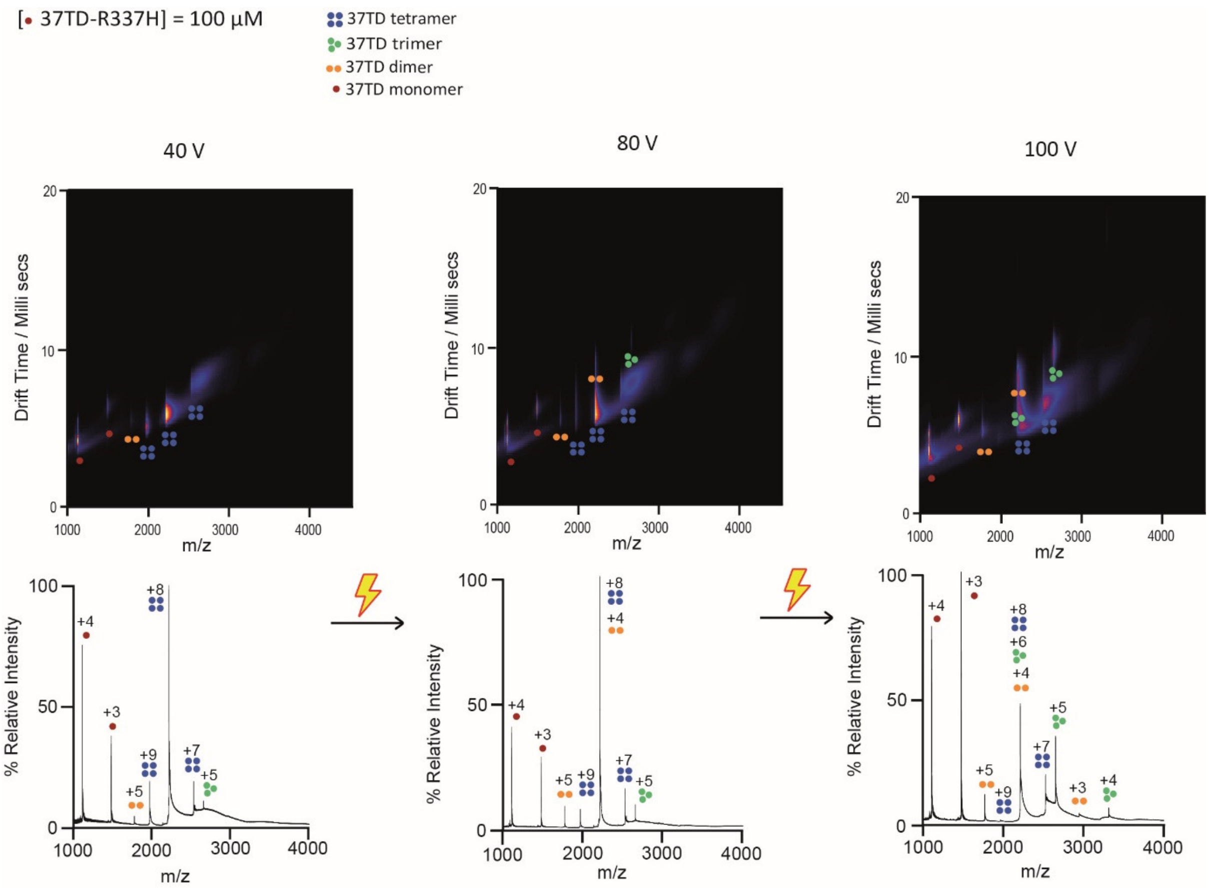

Figure S13. Native Mass Spectrometry and Ion Mobility spectra of 100 μM 37TD-R337H at different cone voltages (40, 80, and 100 V). Non isotope-labelled peptide was used. The concentrations reported refer to the monomer. The sample was dissolved in 200 mM ammonium acetate buffer, pH 7.

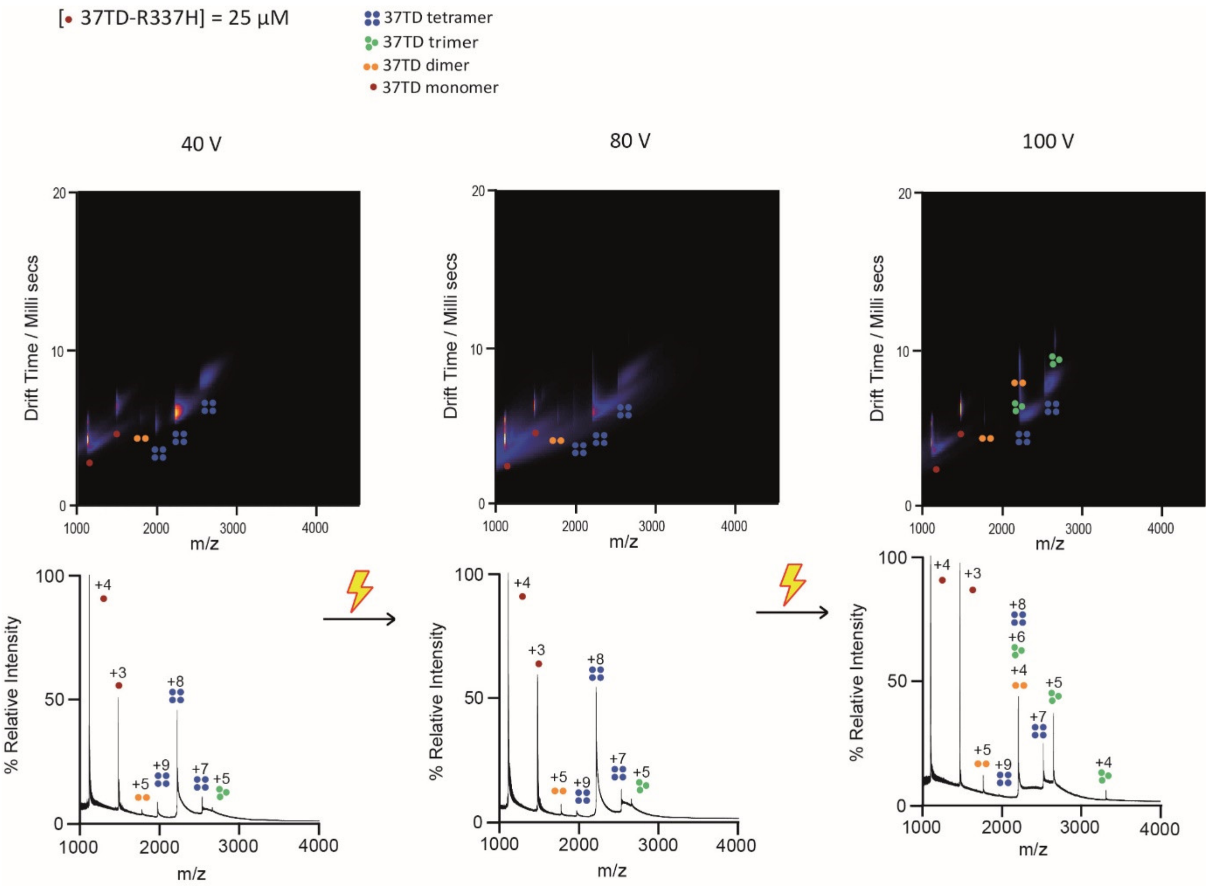

Figure S14. Native Mass Spectrometry and Ion Mobility spectra of 25 µM 37TD-R337H at different cone voltages (40, 80, and 100 V). Non isotope-labelled peptide was used. The concentrations reported refer to the monomer. The sample was dissolved in 200 mM ammonium acetate buffer, pH 7.

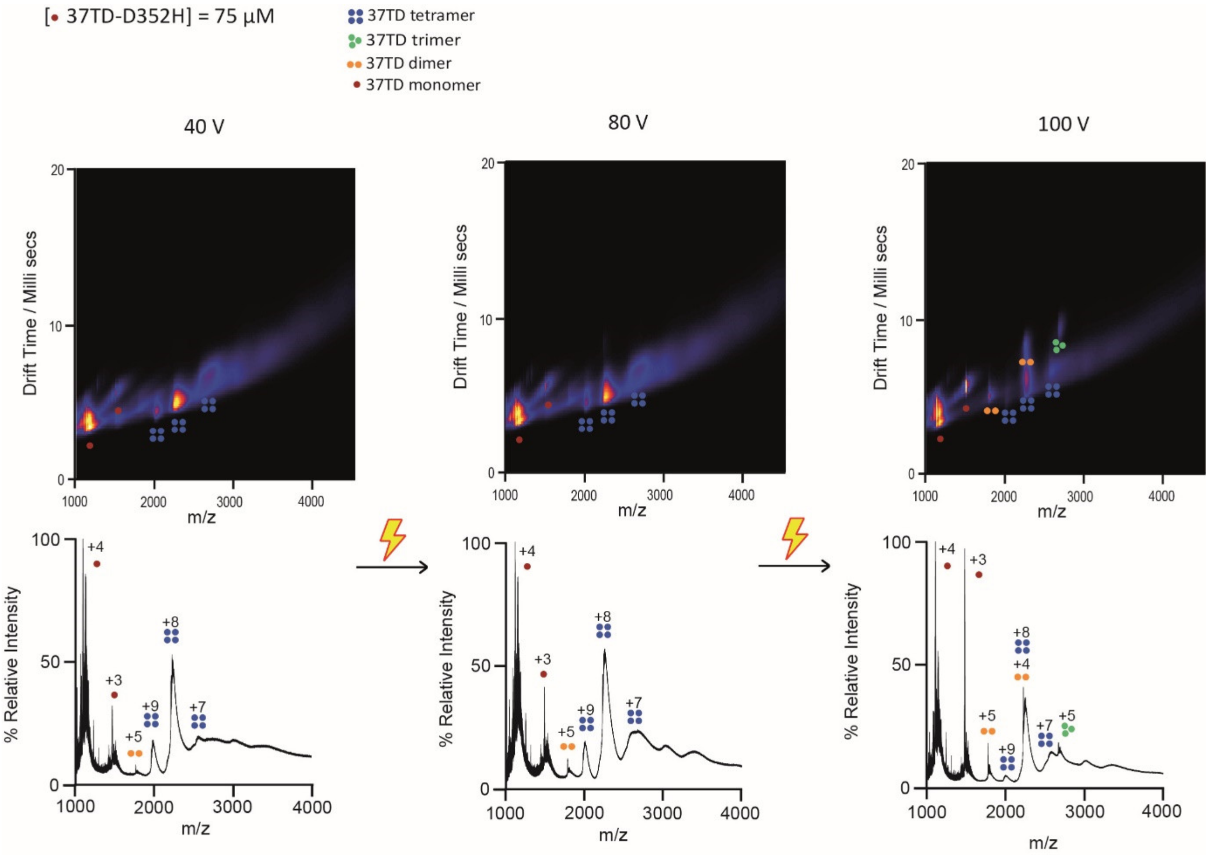

Figure S15. Native Mass Spectrometry and Ion Mobility spectra of 75 µM 37TD-D352H at different cone voltages (40, 80, and 100 V). The concentrations reported refer to the monomer. The sample was dissolved in 200 mM ammonium acetate buffer, pH 7.

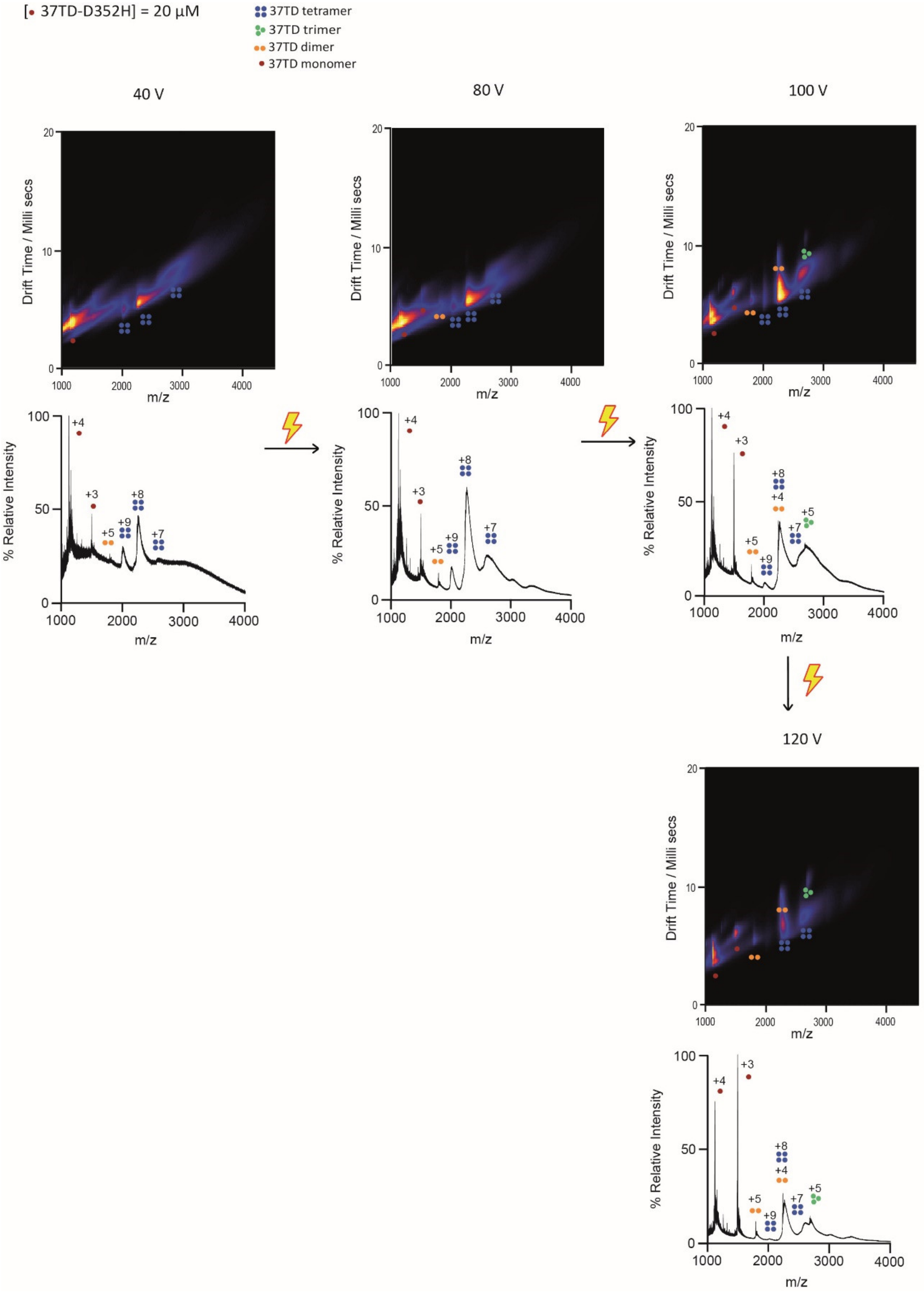

[• 37TD-D352H] = 20 µM
37TD tetramer
37TD trimer
37TD dimer
37TD monomer
40 V
80 V
100 V
120 V
Drift Time / Milli secs
m/z
% Relative Intensity
+4
+3
+5
+9
+8
+7

Figure S16. Native Mass Spectrometry and Ion Mobility spectra of 20 µM 37TD-D352H at different cone voltages (40, 80, and 100 V). The concentrations reported refer to the monomer. The sample was dissolved in 200 mM ammonium acetate buffer, pH 7.

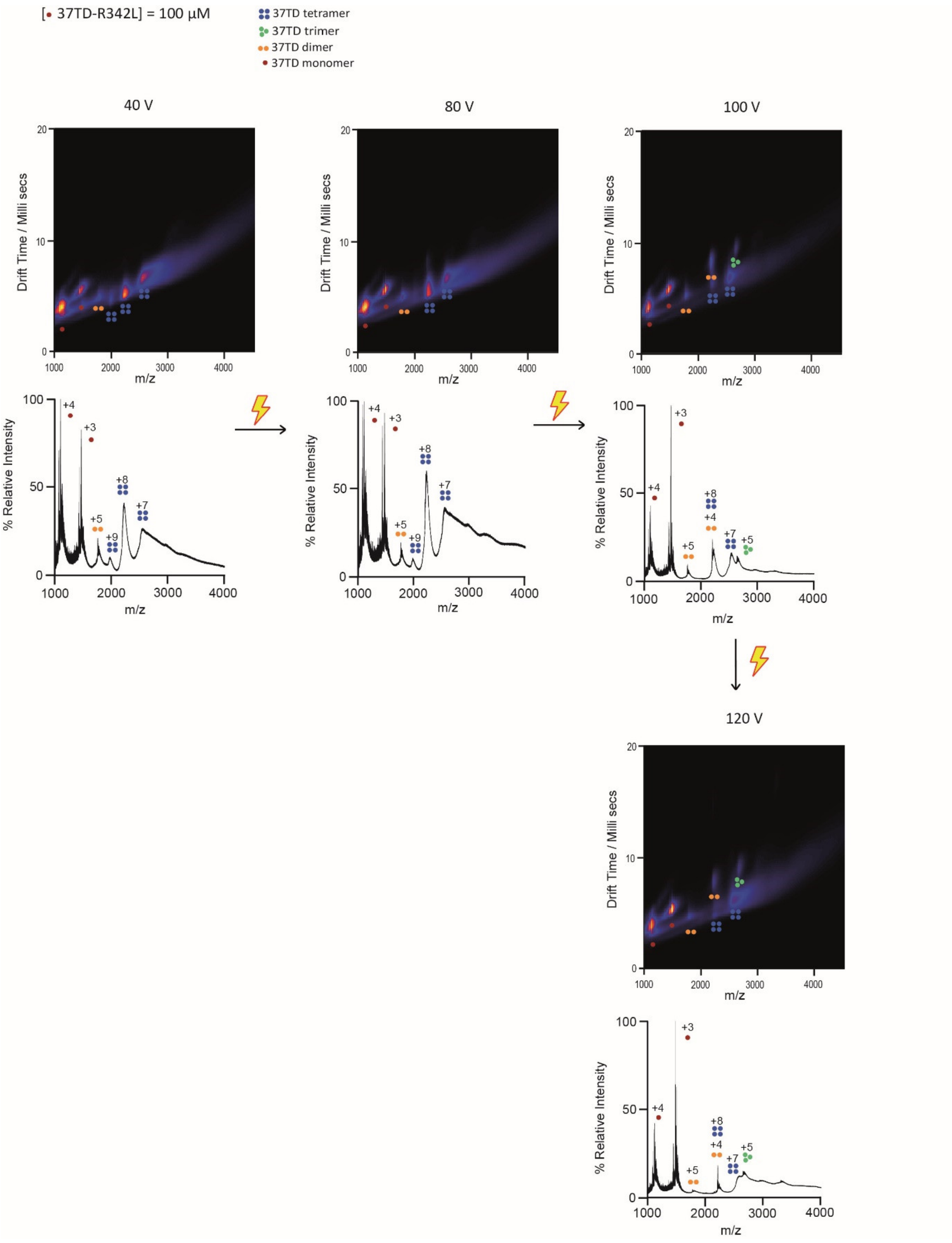

Figure S17. Native Mass Spectrometry and Ion Mobility spectra of 100 µM 37TD-R342L at different cone voltages (40, 80, 100, and 120 V). The concentrations reported refer to the monomer. The sample was dissolved in 200 mM ammonium acetate buffer, pH 7.

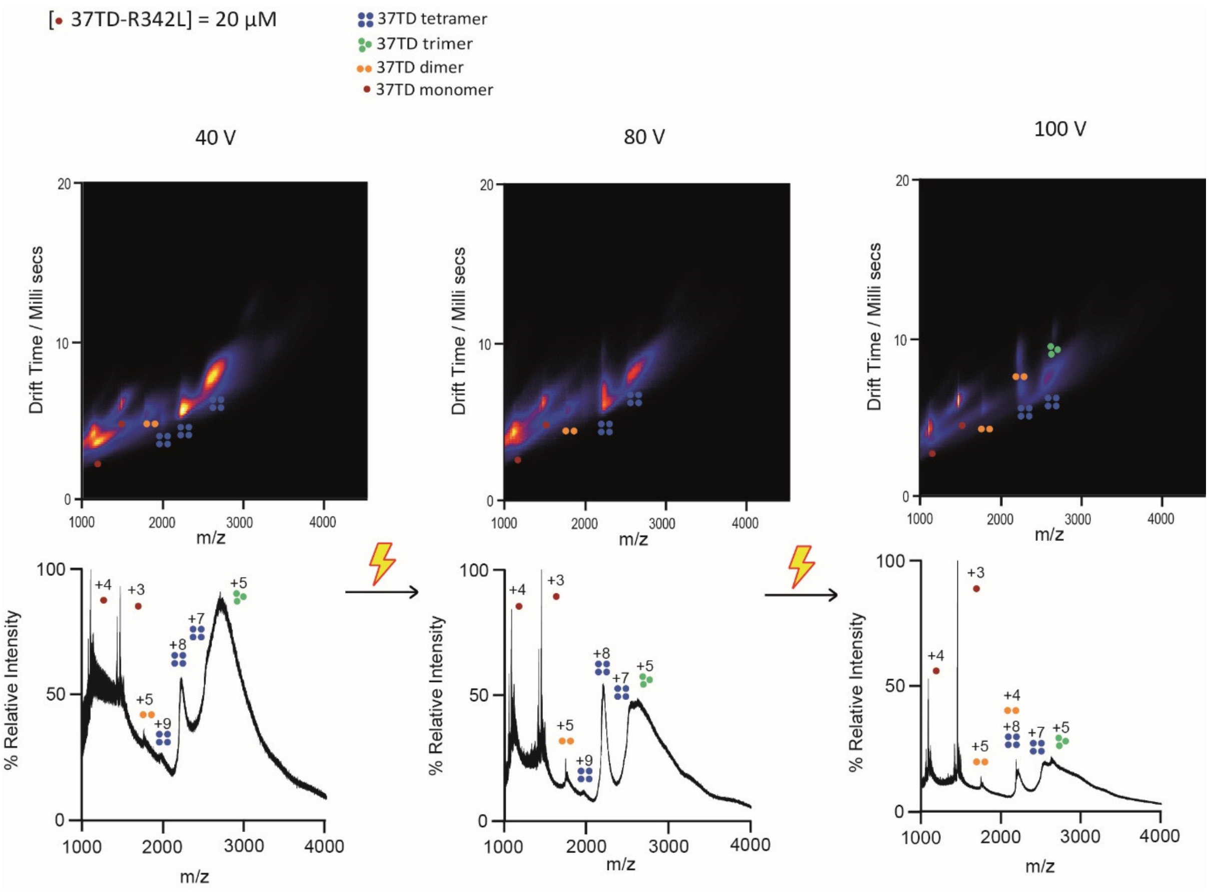

Figure S18. Native Mass Spectrometry and Ion Mobility spectra of 20 µM 37TD-R342L at different cone voltages (40, 80, 100, and 120 V). The concentrations reported refer to the monomer. The sample was dissolved in 200 mM ammonium acetate buffer, pH 7.

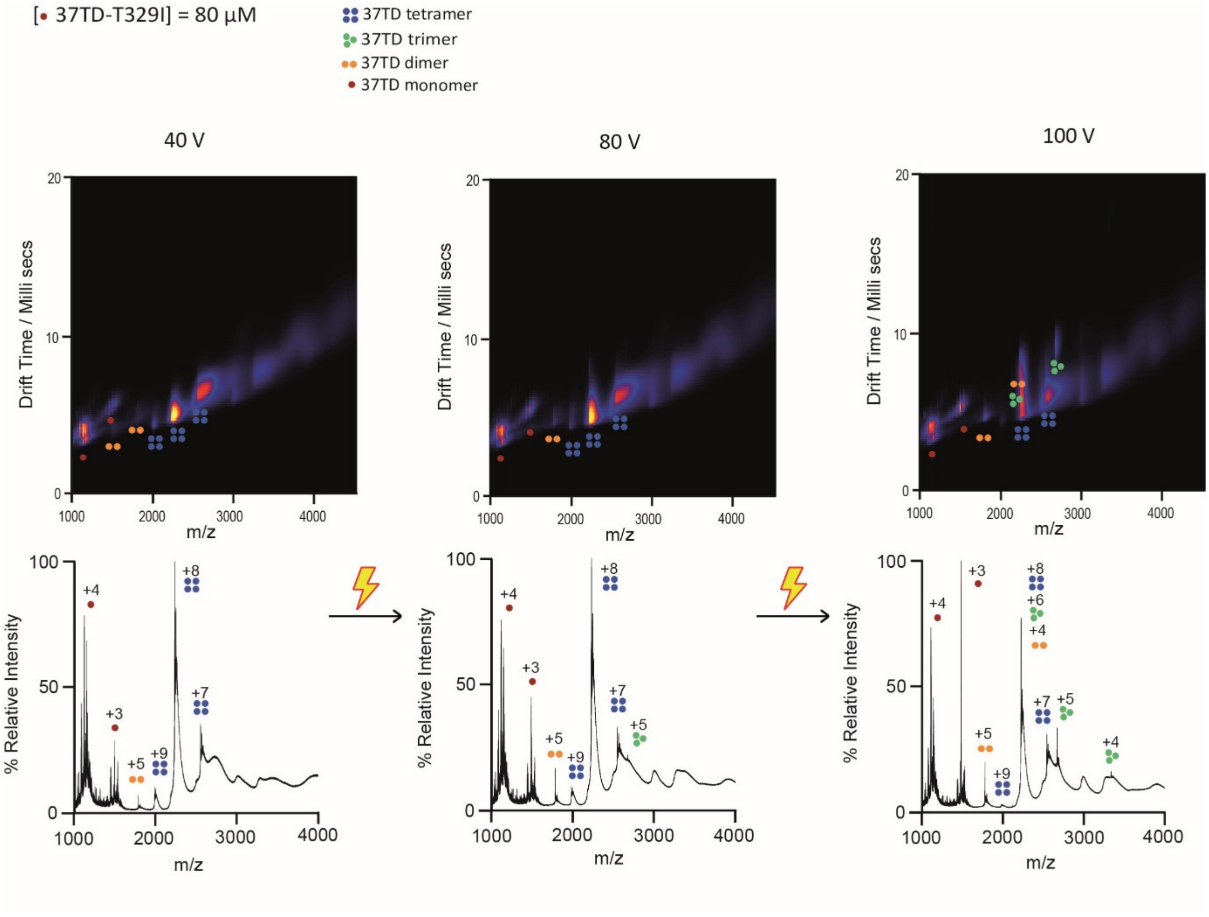

Figure S19. Native Mass Spectrometry and Ion Mobility spectra of 80 µM 37TD-T329I at different cone voltages (40, 80, and 100 V). The concentrations reported refer to the monomer. The sample was dissolved in 200 mM ammonium acetate buffer, pH 7.

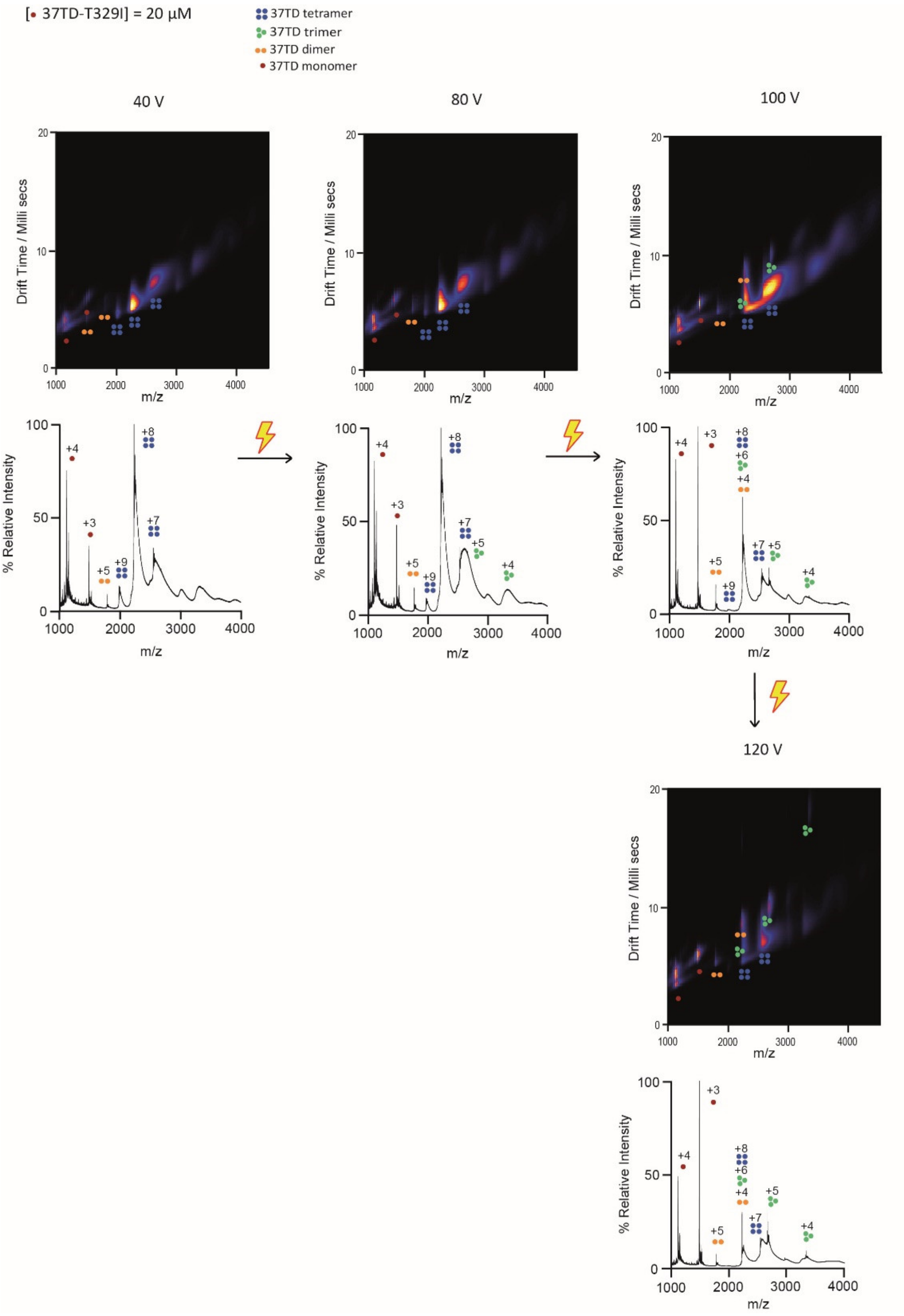

[• 37TD-T329I] = 20 µM
37TD tetramer
37TD trimer
37TD dimer
37TD monomer
40 V
80 V
100 V
120 V
Drift Time / Milli secs
m/z
% Relative Intensity
+4
+3
+5
+9
+8
+7
+6

Figure S20. Native Mass Spectrometry and Ion Mobility spectra of 20 μM 37TD-T329I at different cone voltages (40, 80, and 100 V). The concentrations reported refer to the monomer. The sample was dissolved in 200 mM ammonium acetate buffer, pH 7.

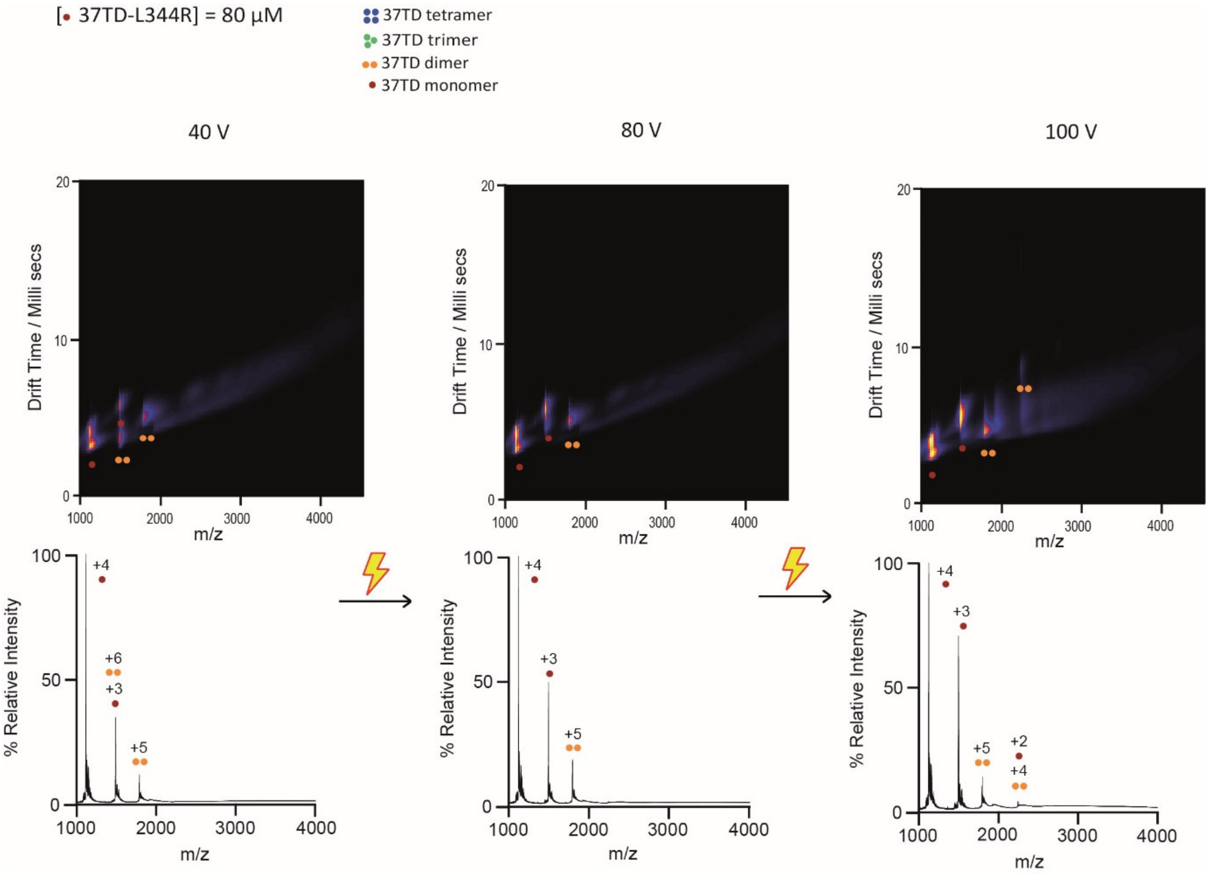

Figure S21. Native Mass Spectrometry and Ion Mobility spectra of 80 μM 37TD-L344R at different cone voltages (40, 80, and 100 V). The concentrations reported refer to the monomer. The sample was dissolved in 200 mM ammonium acetate buffer, pH 7.

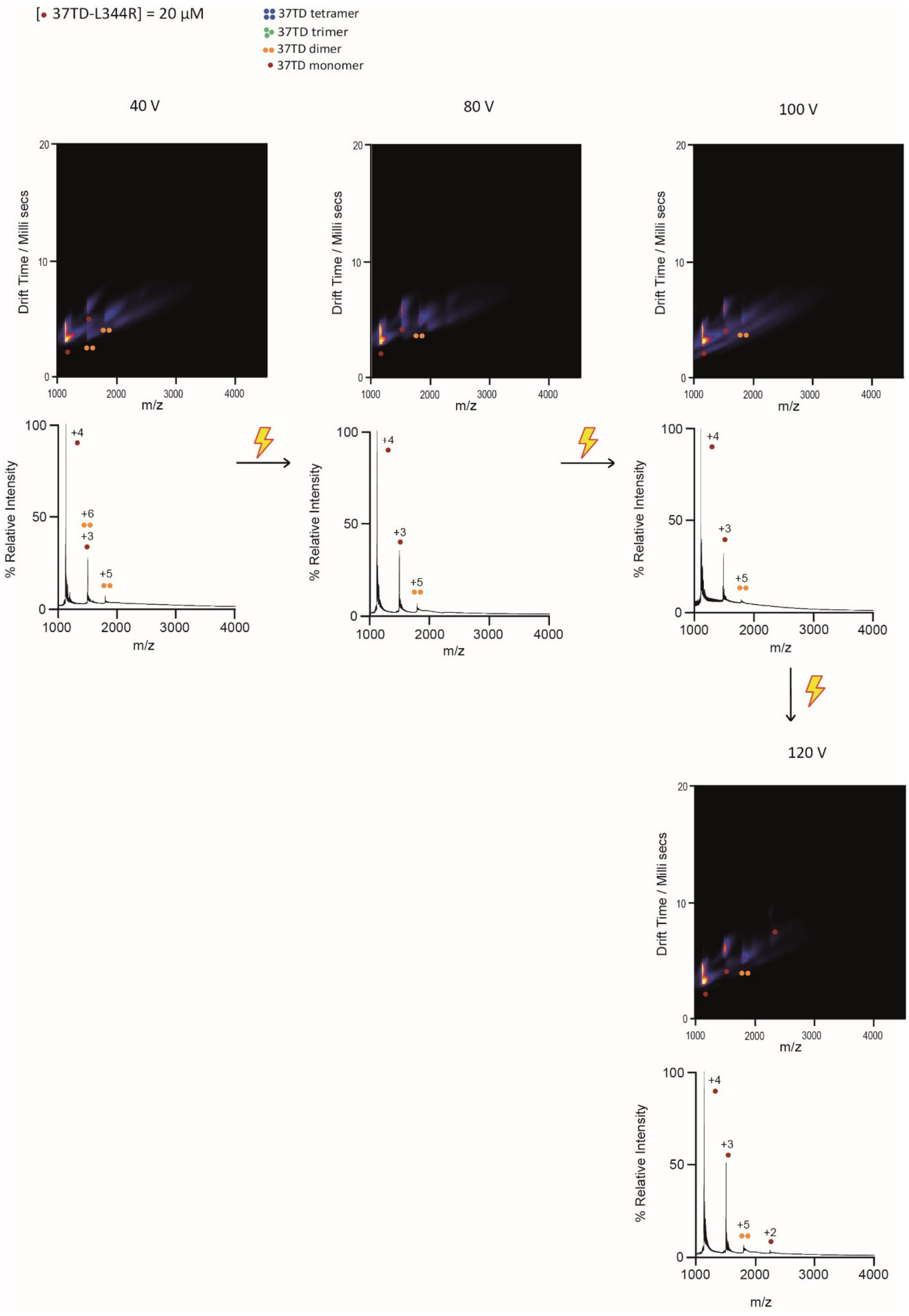

Figure S22. Native Mass Spectrometry and Ion Mobility spectra of 80 μM 37TD-L344R at different cone voltages (40, 80, and 100 V). The concentrations reported refer to the monomer. The sample was dissolved in 200 mM ammonium acetate buffer, pH 7.

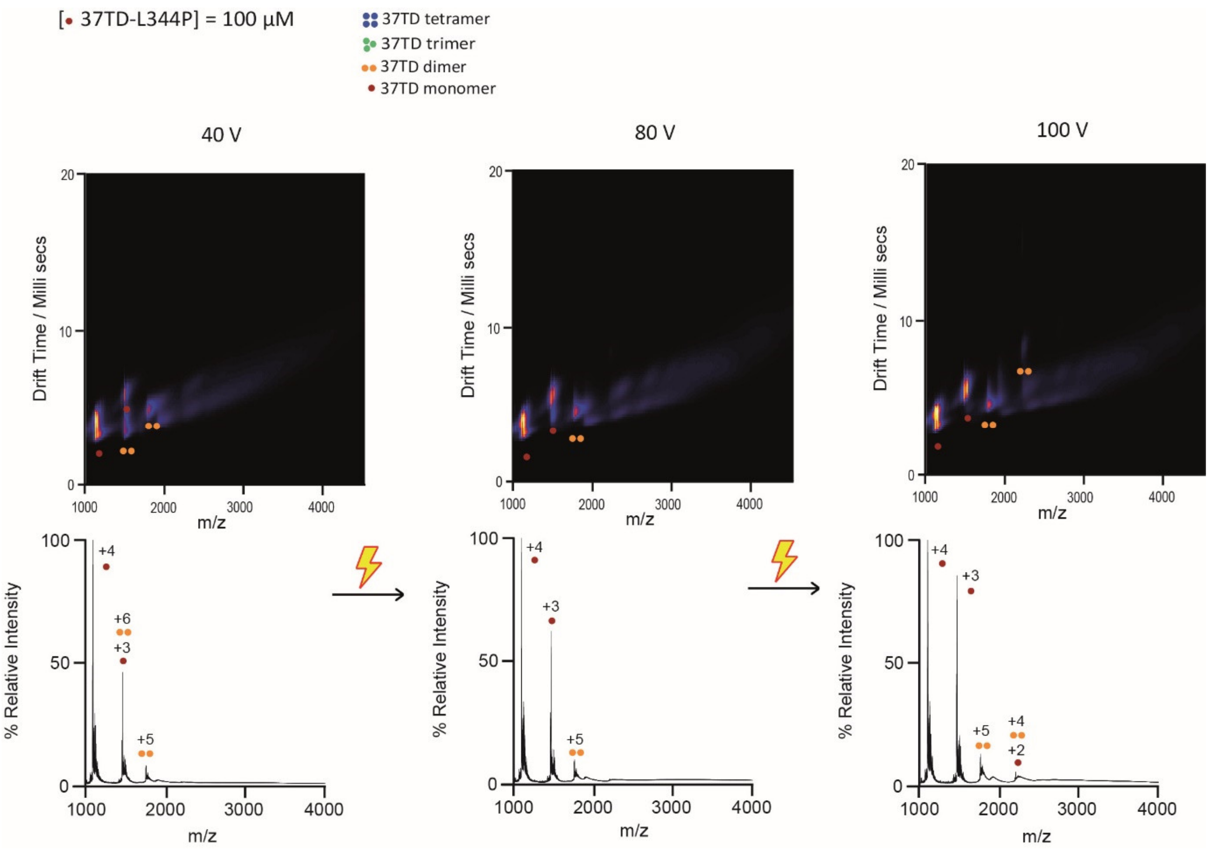

Figure S23. Native Mass Spectrometry and Ion Mobility spectra of 100 µM 37TD-L344P at different cone voltages (40, 80, and 100 V). The concentrations reported refer to the monomer. The sample was dissolved in 200 mM ammonium acetate buffer, pH 7.

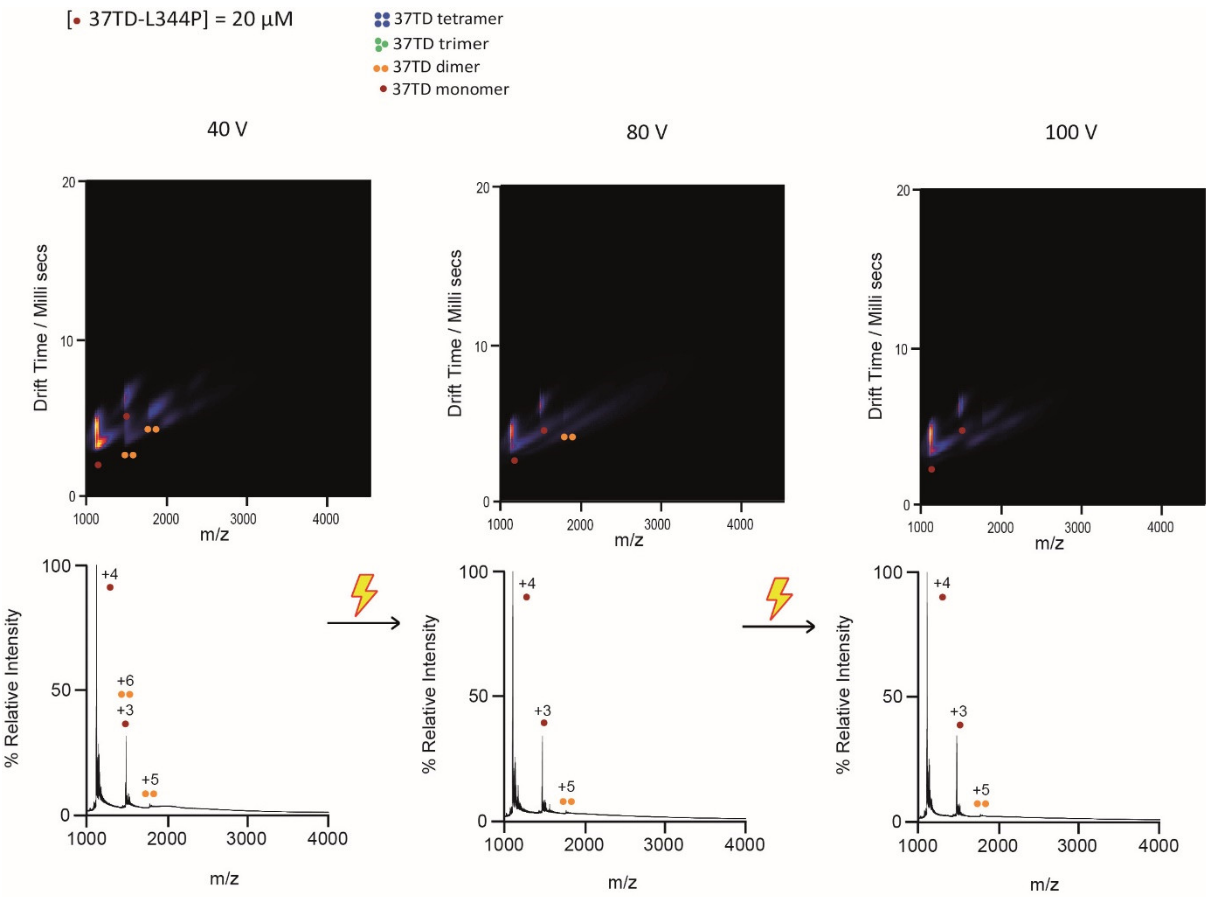

Figure S24. Native Mass Spectrometry and Ion Mobility spectra of 20 µM 37TD-L344P at different cone voltages (40, 80, and 100 V). The concentrations reported refer to the monomer. The sample was dissolved in 200 mM ammonium acetate buffer, pH 7.

37TD-WT

MW 4441,02

		Monomer	Dimer	Trimer	Tetramer	Octamer	Decahexamer
Charge	1	4442,02	8883,04	13324,06	17765,08	35529,16	71057,32
	2	2221,51	4442,02	6662,53	8883,04	17765,08	35529,16
	3	1481,34	2961,68	4442,02	5922,36	11843,72	23686,44
	4	1111,26	2221,51	3331,77	4442,02	8883,04	17765,08
	5	889,20	1777,41	2665,61	3553,82	7106,63	14212,26
	6	741,17	1481,34	2221,51	2961,68	5922,36	11843,72
	7	635,43	1269,86	1904,29	2538,73	5076,45	10151,90
	8	556,13	1111,26	1666,38	2221,51	4442,02	8883,04
	9	494,45	987,89	1481,34	1974,79	3948,57	7896,15
	10	445,10	889,20	1333,31	1777,41	3553,82	7106,63

37TD-R337H

MW 4421,98

		Monomer	Dimer	Trimer	Tetramer
Charge	1	4422,98	8844,96	13266,94	17688,92
	2	2211,99	4422,98	6633,97	8844,96
	3	1474,99	2948,99	4422,98	5896,97
	4	1106,50	2211,99	3317,49	4422,98
	5	885,40	1769,79	2654,19	3538,58
	6	738,00	1474,99	2211,99	2948,99
	7	632,71	1264,42	1896,13	2527,85
	8	553,75	1106,50	1659,24	2211,99
	9	492,33	983,66	1474,99	1966,32
	10	443,20	885,40	1327,59	1769,79

37TD-T329I

MW 4454,077

		Monomer	Dimer	Trimer	Tetramer
Charge	1	4455,08	8909,15	13363,23	17817,31
	2	2228,04	4455,08	6682,12	8909,15
	3	1485,69	2970,38	4455,08	5939,77
	4	1114,52	2228,04	3341,56	4455,08
	5	891,82	1782,63	2673,45	3564,26
	6	743,35	1485,69	2228,04	2970,38
	7	637,30	1273,59	1909,89	2546,19
	8	557,76	1114,52	1671,28	2228,04
	9	495,90	990,79	1485,69	1980,59
	10	446,41	891,82	1337,22	1782,63

37TD-D352H

MW 4464,08

		Monomer	Dimer	Trimer	Tetramer
Charge	1	4465,08	8929,15	13393,23	17857,30
	2	2233,04	4465,08	6697,11	8929,15
	3	1489,03	2977,05	4465,08	5953,10
	4	1117,02	2233,04	3349,06	4465,08
	5	893,82	1786,63	2679,45	3572,26
	6	745,01	1489,03	2233,04	2977,05
	7	638,73	1276,45	1914,18	2551,90
	8	559,01	1117,02	1675,03	2233,04
	9	497,01	993,02	1489,03	1985,03
	10	447,41	893,82	1340,22	1786,63

37TD-L344R

MW 4485,051

		Monomer	Dimer	Trimer	Tetramer
Charge	1	4486,05	8971,10	13456,15	17941,20
	2	2243,53	4486,05	6728,58	8971,10
	3	1496,02	2991,03	4486,05	5981,07
	4	1122,26	2243,53	3364,79	4486,05
	5	898,01	1795,02	2692,03	3589,04
	6	748,51	1496,02	2243,53	2991,03
	7	641,72	1282,44	1923,16	2563,89
	8	561,63	1122,26	1682,89	2243,53
	9	499,34	997,68	1496,02	1994,36
	10	449,51	898,01	1346,52	1795,02

37TD-R342L

MW 4399,00

		Monomer	Dimer	Trimer	Tetramer
Charge	1	4400,00	8798,99	13197,99	17596,98
	2	2200,50	4400,00	6599,49	8798,99
	3	1467,33	2933,66	4400,00	5866,33
	4	1100,75	2200,50	3300,25	4400,00
	5	880,80	1760,60	2640,40	3520,20
	6	734,17	1467,33	2200,50	2933,66
	7	629,43	1257,86	1886,28	2514,71
	8	550,87	1100,75	1650,62	2200,50
	9	489,78	978,55	1467,33	1956,11
	10	440,90	880,80	1320,70	1760,60

37TD-L344P

MW 4425,98

		Monomer	Dimer	Trimer	Tetramer
Charge	1	4426,98	8852,96	13278,94	17704,92
	2	2213,99	4426,98	6639,97	8852,96
	3	1476,33	2951,65	4426,98	5902,31
	4	1107,50	2213,99	3320,49	4426,98
	5	886,20	1771,39	2656,59	3541,78
	6	738,66	1476,33	2213,99	2951,65
	7	633,28	1265,57	1897,85	2530,13
	8	554,25	1107,50	1660,74	2213,99
	9	492,78	984,55	1476,33	1968,10
	10	443,60	886,20	1328,79	1771,39

Table S3. Masses over charges (m/z) of the 37TDs in monomeric, dimeric, trimeric, and tetrameric states.

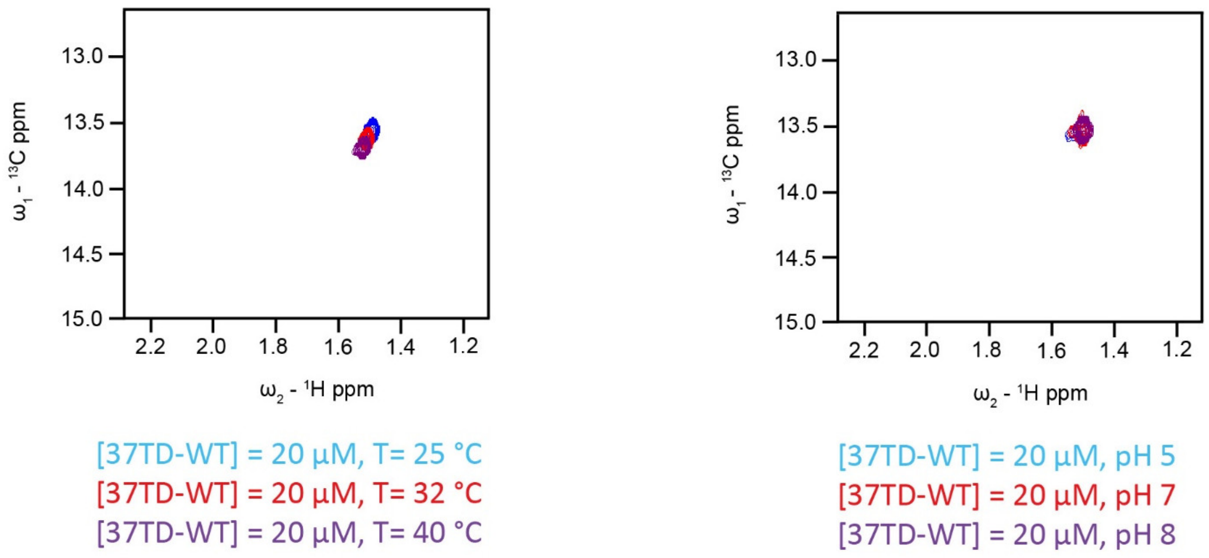

Figure S25. 2D ^{1}H,^{13}C HSQC spectra of 37TD-WT at different pH and temperature. The concentrations refer to the monomer. All the samples were dissolved in D_2O, pH 7, and all the spectra recorded at 25 °C unless otherwise indicated.

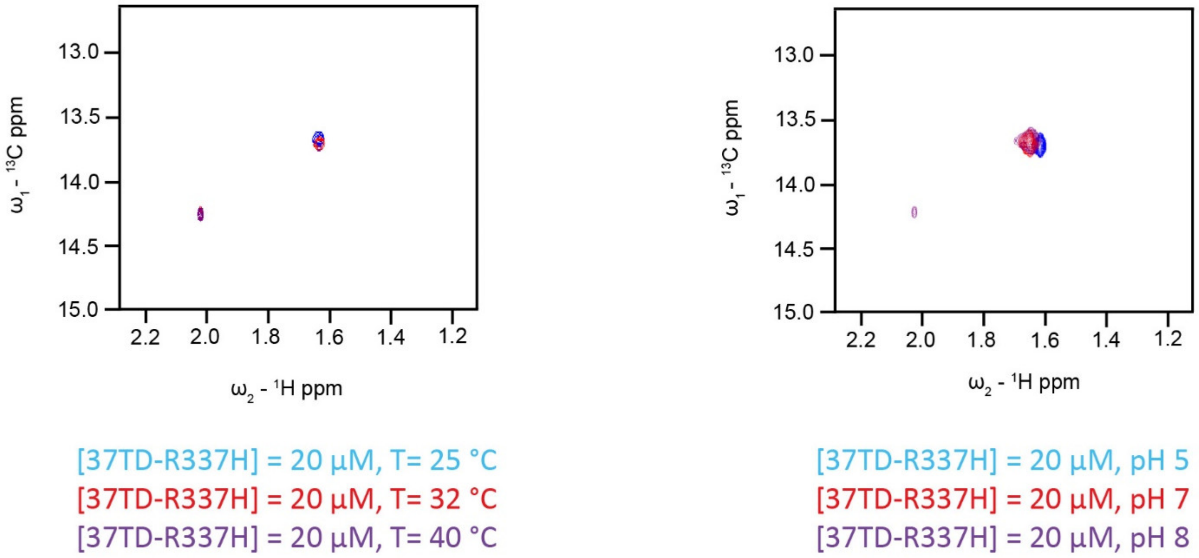

Figure S26. 2D ^{1}H,^{13}C HSQC spectra of 37TD-R337H at different pH and temperature. The concentrations refer to the monomer. All the samples were dissolved in D_2O, pH 7, and all the spectra recorded at 25 °C unless otherwise indicated.

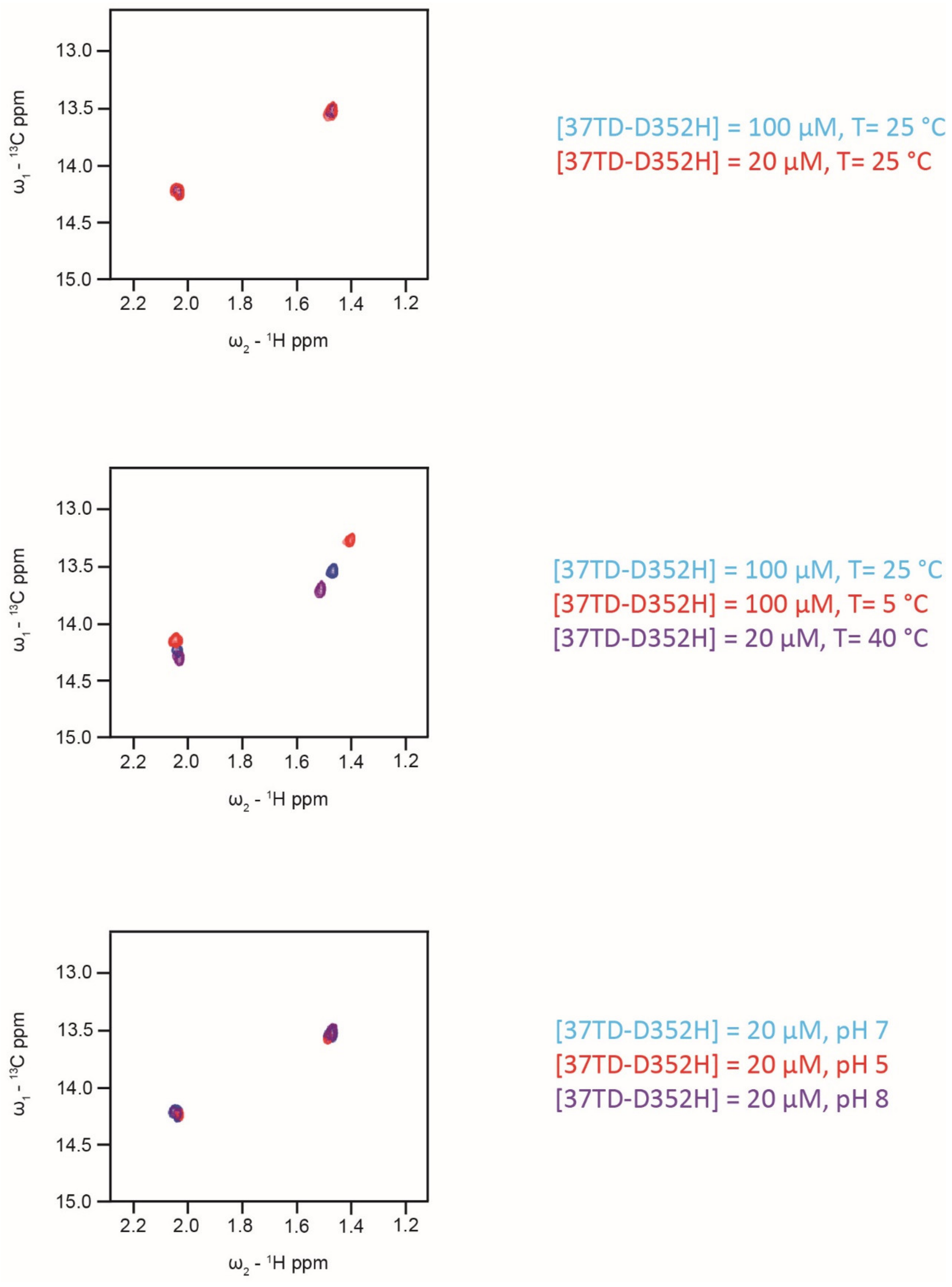

Figure S27. 2D ^1H,^{13}C HSQC spectra of 37TD-D352H at different sample concentration, temperature and pH. The concentrations refer to the monomer. All the samples were dissolved in D_2O, pH 7, and all the spectra recorded at 25 ^oC unless otherwise indicated.

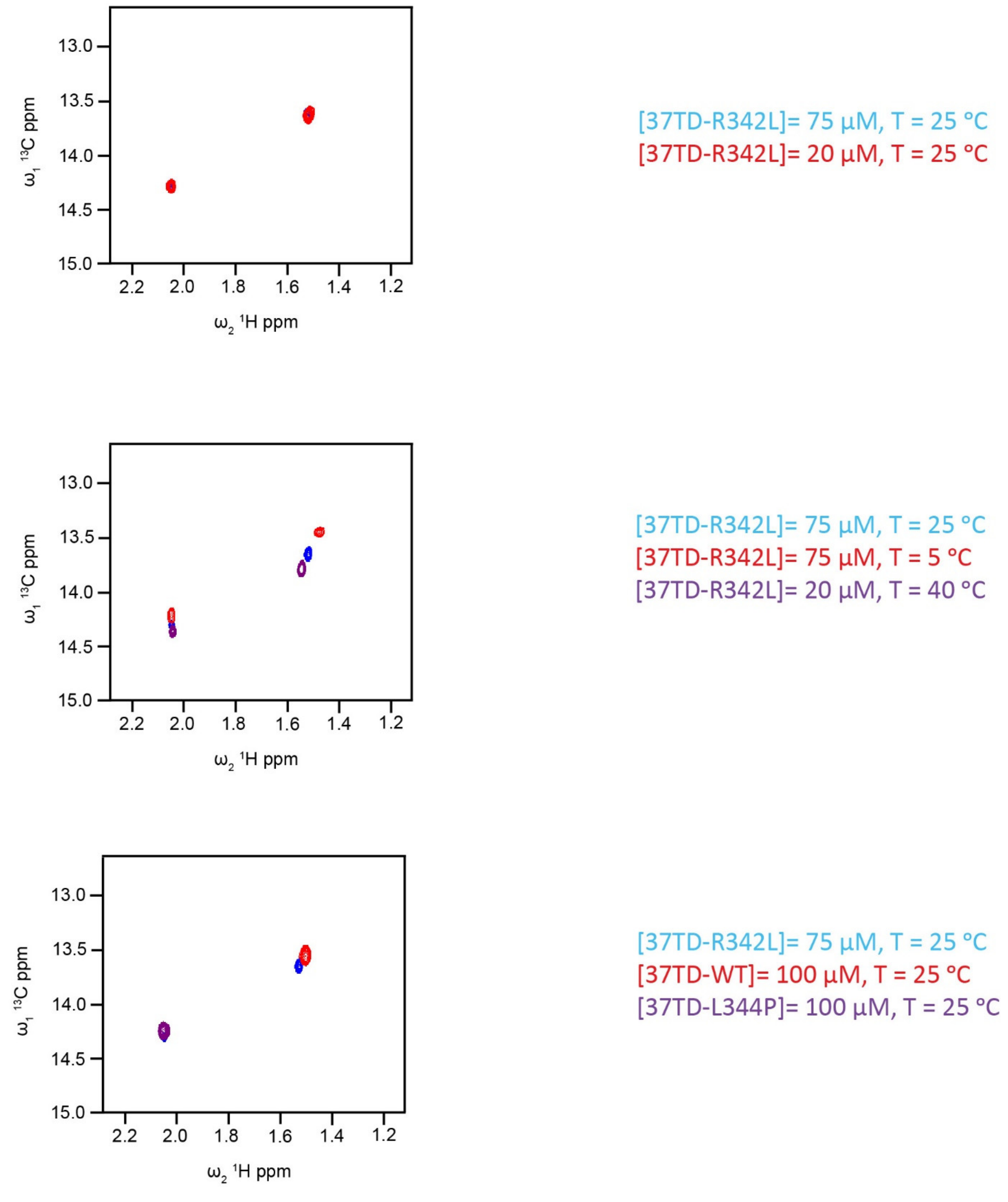

Figure S28. 2D ^{1}H,^{13}C HSQC spectra of 37TD-R342L at different sample concentration and temperature. The concentrations refer to the monomer. All the samples were dissolved in D_2O, pH 7, and all the spectra recorded at 25 °C unless otherwise indicated.

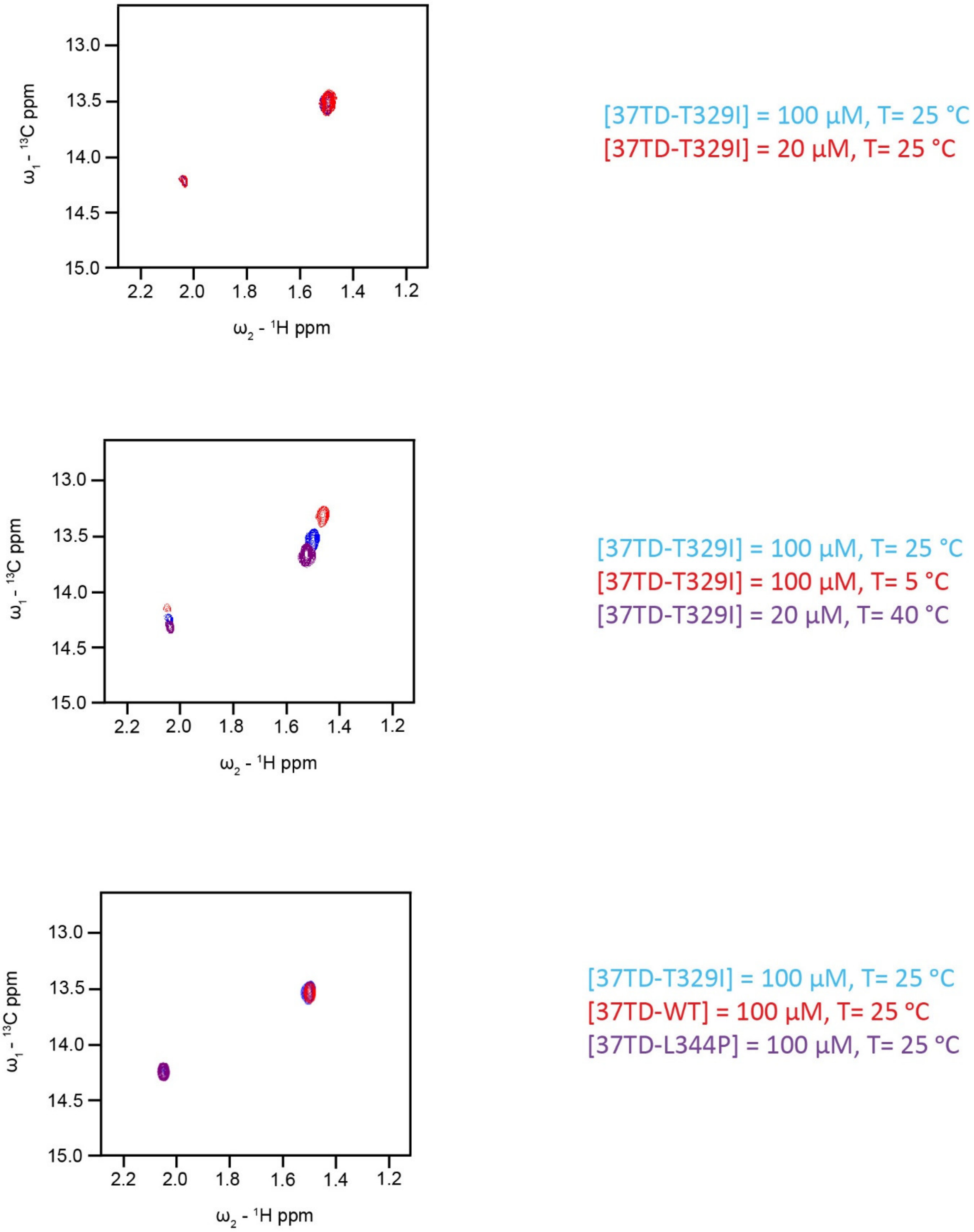

Figure S29. 2D ^{1}H,^{13}C HSQC spectra of 37TD-T329I at different sample concentration and temperature. The concentrations refer to the monomer. All the samples were dissolved in D_2O, pH 7, and all the spectra recorded at 25 ^{o}C unless otherwise indicated.

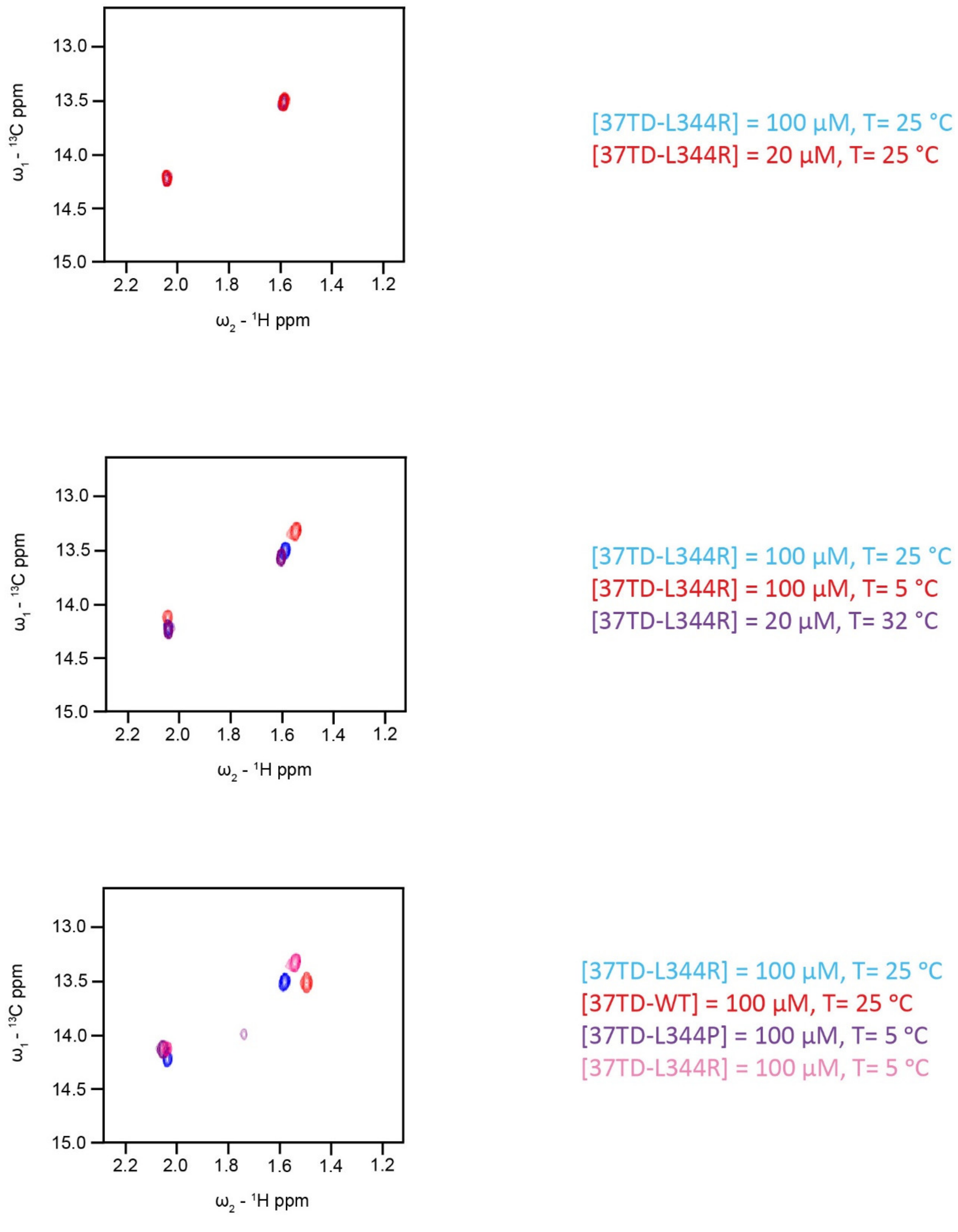

Figure S30. 2D ^{1}H,^{13}C HSQC spectra of 37TD-L344R at different sample concentration and temperature. The concentrations refer to the monomer. All the samples were dissolved in D_2O, pH 7, and all the spectra recorded at 25 ^{o}C unless otherwise indicated.

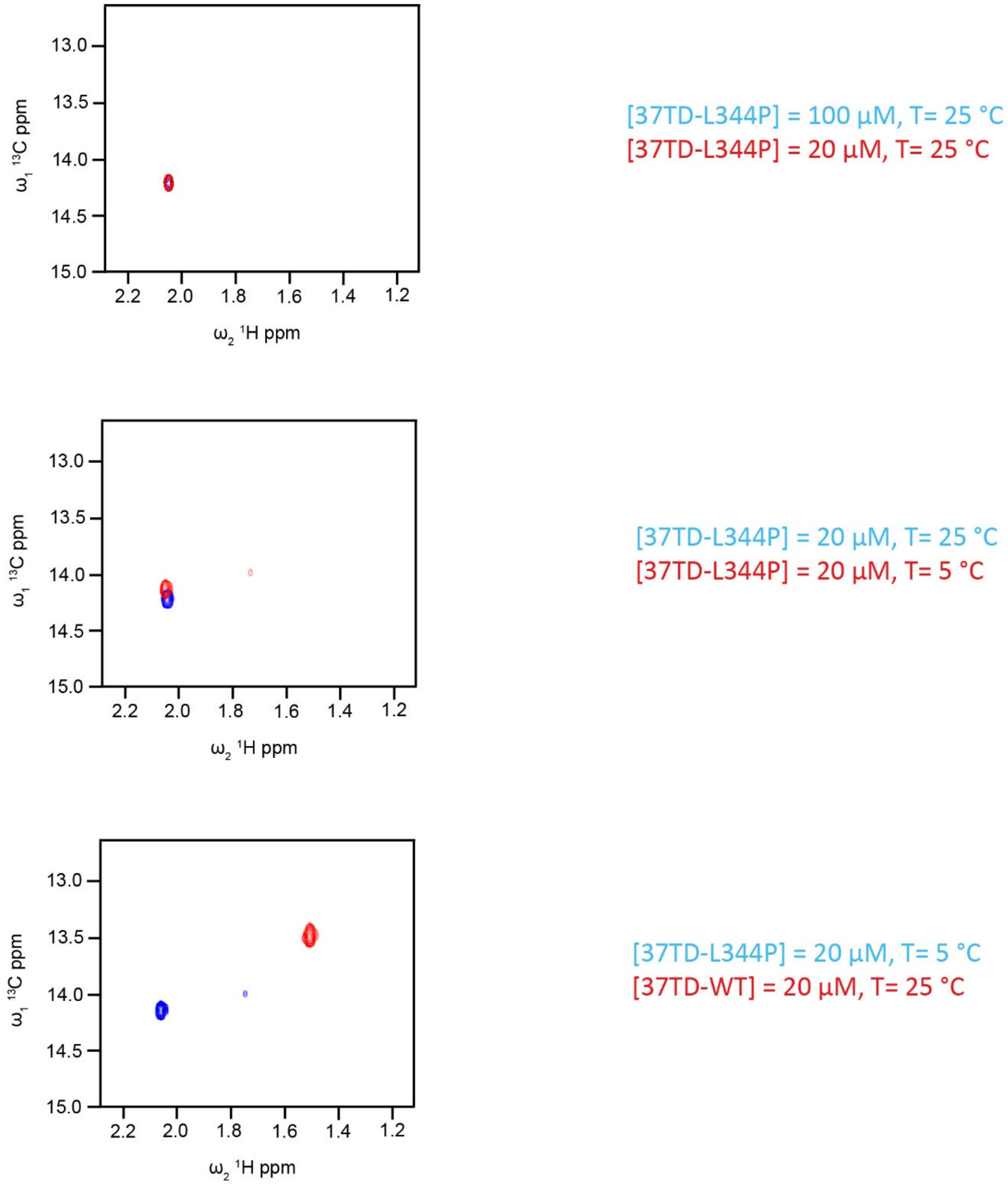

Figure S31. 2D ^{1}H,^{13}C HSQC spectra of 37TD-L344P at different sample concentration and temperature. The concentrations refer to the monomer. All the samples were dissolved in D_2O, pH 7, and all the spectra recorded at 25 °C unless otherwise indicated.

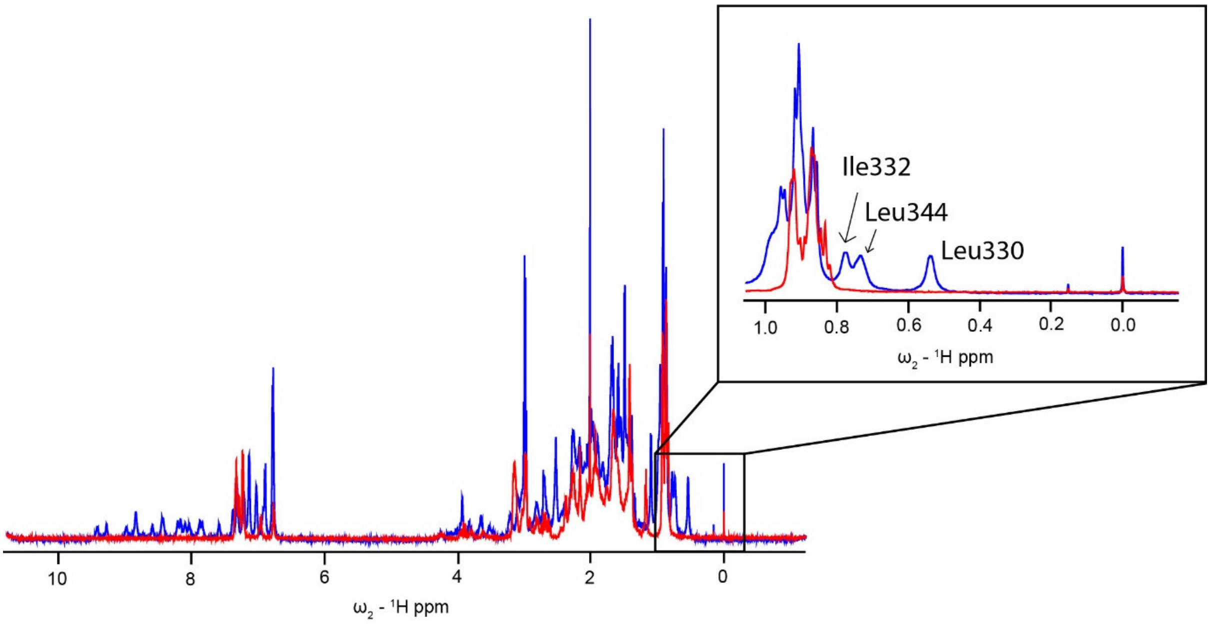

Figure S32. 1D ^{1}H spectra of 37TD-WT (blue) and 37TD-L344P (red) dissolved in D_2O, pH 7, at a monomer concentration of 100 μM. The spectra are recorded at 25 ^{o}C.

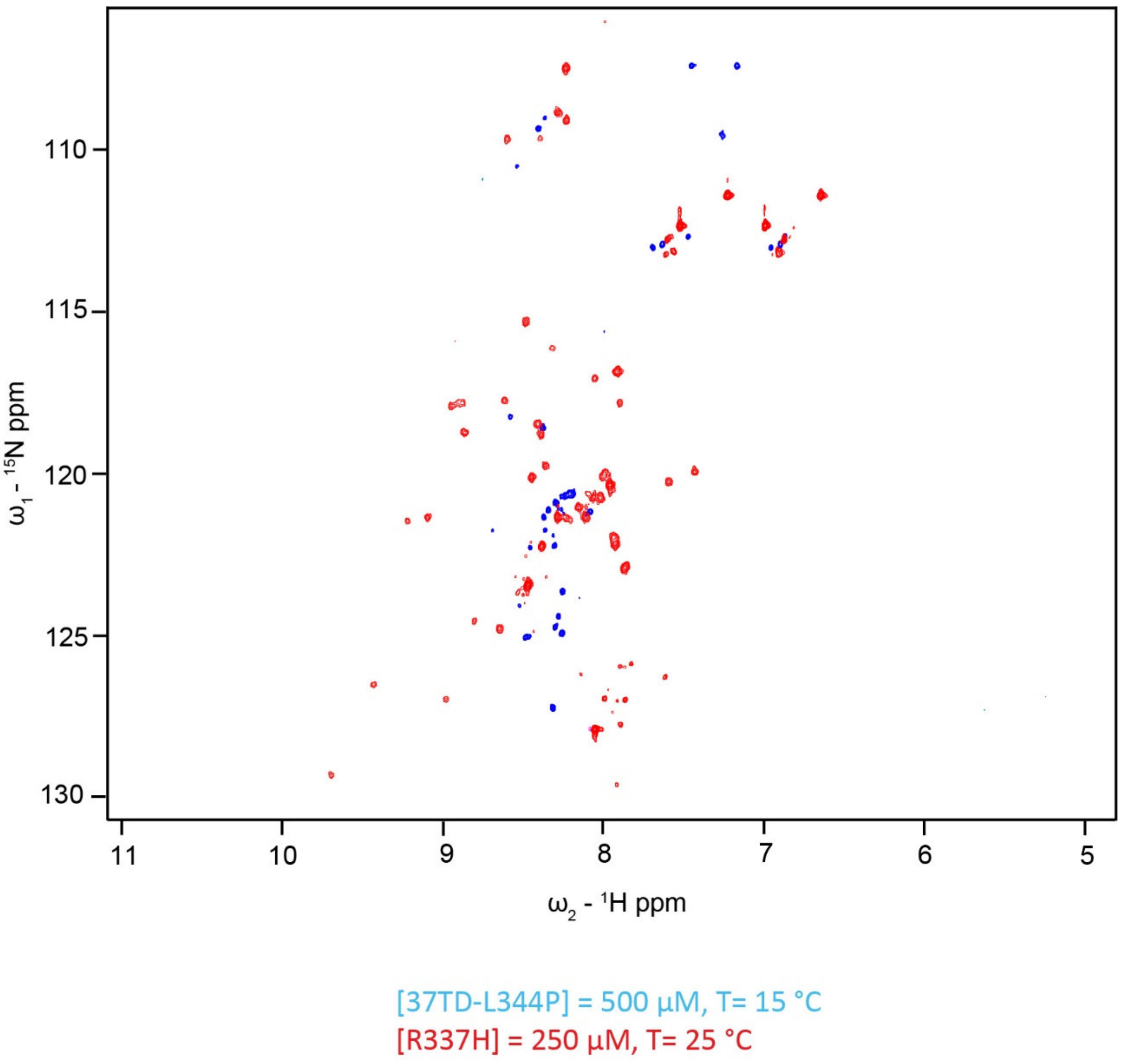

Figure S33. In blue, ^{1}H,^{15}N HSQC spectrum of 37TD-L344P dissolved in D_2O, pH 7. The signals of the spectrum are due to the natural abundance of ^{15}N. For comparison, in red, ^{1}H,^{15}N HSQC spectrum of a reference R337H, res 311-367, expressed in *E. coli* and enriched in ^{15}N [52]. R337H was dissolved in 10 mM sodium phosphate buffer, pH 7. The concentrations refer to the monomer.

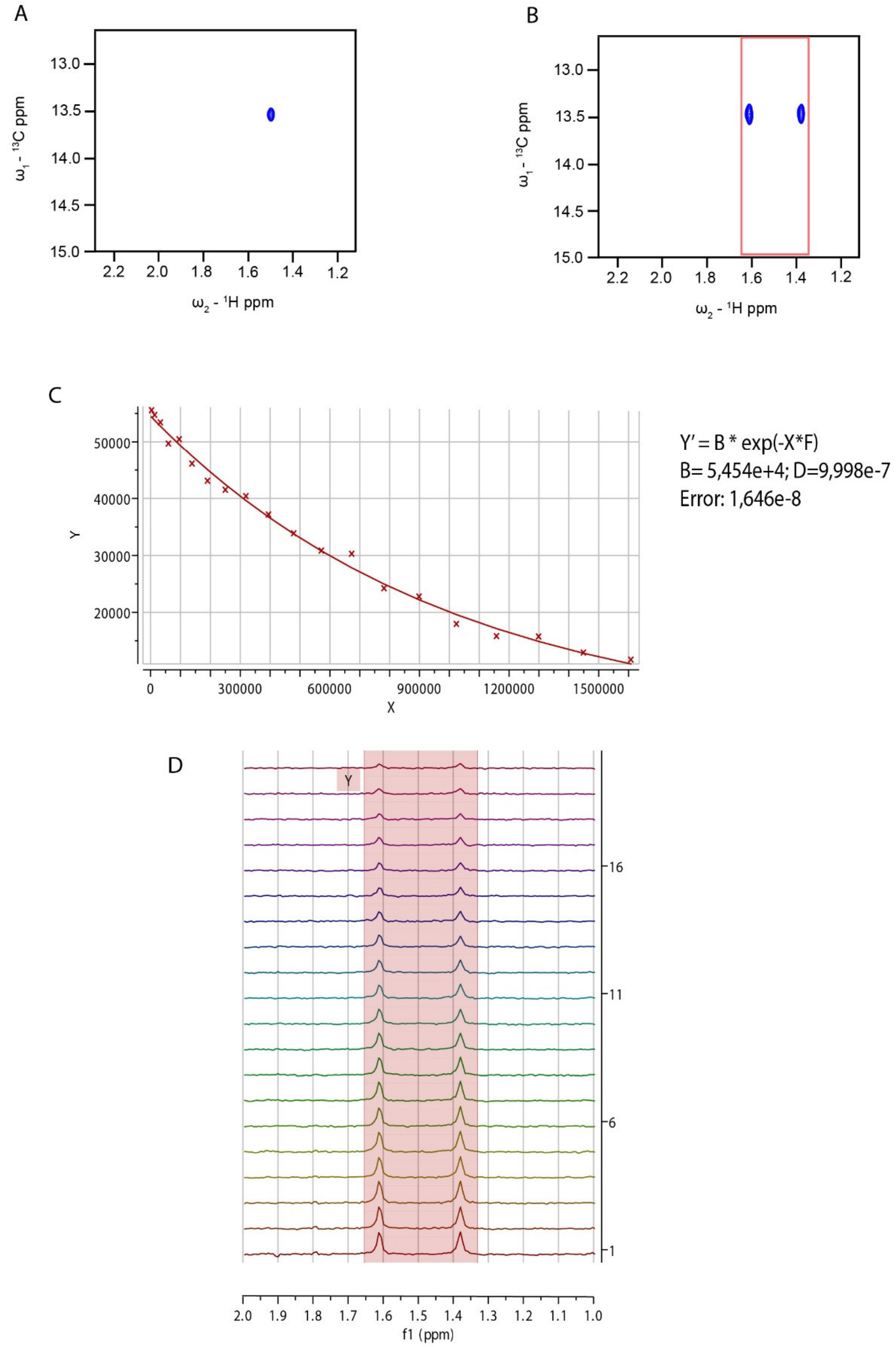

Figure S34. X-STE NMR diffusion of 37TD-WT monitoring the size of the species containing ^{13}C-attached ^{1}H nuclei. (A) 2D ^{1}H,^{13}C-HSQC. (B) ^{13}C-coupled 2D ^{1}H,^{13}C-HSQC illustrating the splitting of the Met340 $^{1}H^{\varepsilon}$ signal ($^{1}J_{HC}$ ≈140 Hz). (C,D)) X-STE NMR diffusion experiments. The decay in signal intensity of the Met340 $^{1}H^{\varepsilon}$ signal (panel D) was fitted to a mono exponential function to obtain the coefficient diffusion. X-STE experiments were acquired without decoupling and the Met340 $^{1}H^{\varepsilon}$ signal splits into a doublet ($^{1}J_{HC}$ ≈140 Hz). The spectra were recorded at 25 °C using a monomer concentration of 100 μM (D_2O, pH 7).

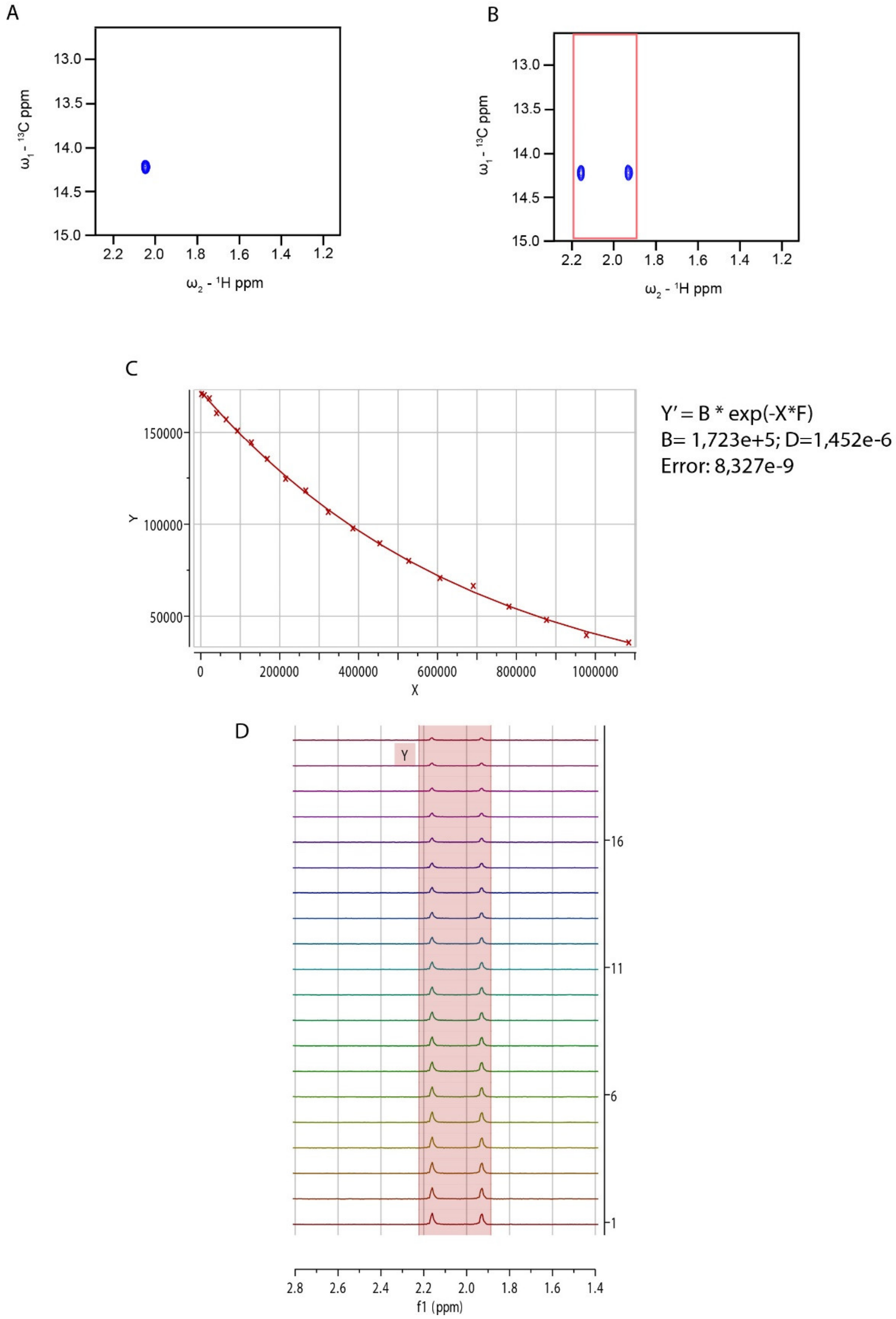

Figure S35. X-STE NMR diffusion of 37TD-L344P monitoring the size of the species containing ^{13}C-attached ^{1}H nuclei. (A) 2D ^{1}H,^{13}C-HSQC. (B) ^{13}C-coupled 2D ^{1}H,^{13}C-HSQC illustrating the splitting of the Met340 ^{1}H$^{\varepsilon}$ signal ($^{1}J_{HC}$ ≈140 Hz). (C,D)) X-STE NMR diffusion experiments. The decay in signal intensity of the Met340 ^{1}H$^{\varepsilon}$ signal (panel D) was fitted to a mono exponential function to obtain the coefficient diffusion. X-STE experiments were acquired without decoupling and the Met340 ^{1}H$^{\varepsilon}$ signal splits into a doublet ($^{1}J_{HC}$ ≈140 Hz). The spectra were recorded at 25 ^{o}C using a monomer concentration of 100 μM (D_2O, pH 7).

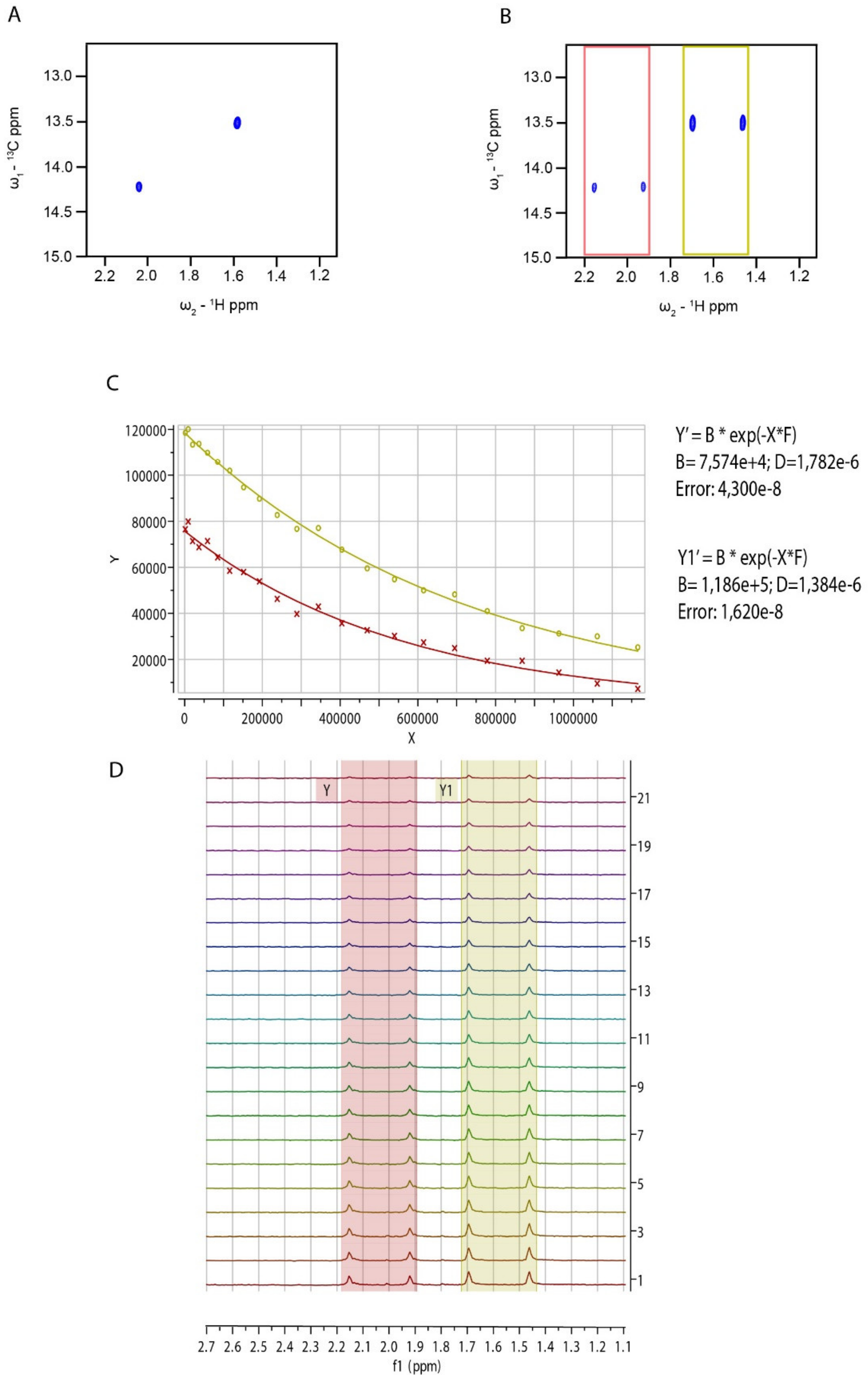

Figure S36. X-STE NMR diffusion of 37TD-L344R monitoring the size of the species containing ^{13}C-attached ^{1}H nuclei. (A) 2D ^{1}H,^{13}C-HSQC. (B) ^{13}C-coupled 2D ^{1}H,^{13}C-HSQC illustrating the splitting of the Met340 $^{1}H^{\varepsilon}$ signal ($^{1}J_{HC}$ ≈140 Hz). (C,D)) X-STE NMR diffusion experiments. The decay in signal intensity of the Met340 $^{1}H^{\varepsilon}$ signal (panel D) was fitted to a mono exponential function to obtain the coefficient diffusion. X-STE experiments were acquired without decoupling and the Met340 $^{1}H^{\varepsilon}$ signal splits into a doublet ($^{1}J_{HC}$ ≈140 Hz). The spectra were recorded at 25 °C using a monomer concentration of 100 μM (D_2O, pH 7).
